# Supplementary material for: Bi-allelic ATG12 variants impair autophagy and cause a neurodevelopmental disorder
Source: Am J Hum Genet. 2026 Mar 26;113(5):1090–107. doi: 10.1016/j.ajhg.2026.03.002 (PMC13277686; doi:10.1016/j.ajhg.2026.03.002)
Supplement: Document S1. Figures S1 and S2 and Tables S1–S5 [file mmc1.pdf]

The American Journal of Human Genetics, Volume *113*

Supplemental information

Bi-allelic *ATG12* variants impair autophagy and cause a neurodevelopmental disorder

James Lambton, Shotaro Asano, Yuxiang Huang, Fumi Suomi, Tomoya Eguchi, Cassidy Petree, Kevin Huang, Magali Prigent, Aliza Imam, Thomas J. McCorvie, Daniel Warren, Emma Hobson, Helen McCullagh, Doriana Misceo, Anna Bjerre, Marie F. Smeland, Claus Klingenberg, Eirik Frengen, Swati Naik, Gavin Ryan, Annapurna Sudarsanam, Katherine Foster, Pradeep Vasudevan, Rajib Samanta, Fatima Rahman, Shazia Maqbool, Vrajesh Udani, Stephanie Efthymiou, Henry Houlden, Robert McFarland, Jack J. Collier, Reza Maroofian, Wyatt W. Yue, Gaurav K. Varshney, Daniel J. Klionsky, Renaud Legouis, Thomas G. McWilliams, Noboru Mizushima, Monika Oláhová, Charlotte L. Alston, and Robert W. Taylor

Supplemental materials

Clinical Descriptions

Family 1

S1 (Family 1, **Figure 1A**), a female infant, was the first child born to non-consanguineous white European parents following *in vitro* fertilization (IVF) for unexplained infertility. Born at term, by normal vaginal delivery following an uneventful pregnancy, she did not require resuscitation and weighed 3.225 kg (25th-50th centile). Newborn examination was normal with the exception of overlapping 4th and 5th toes bilaterally. On day 3 of life, feeding difficulties, excessive weight loss and jaundice were noted. Examination revealed hypotonia and apnoea when feeding. Supplemental oxygen and nasogastric tube feeding were commenced, and a subsequent swallowing assessment confirmed a poorly coordinated swallow.

Axial hypotonia with excessive head lag remained a prominent feature on neurological review at 6 weeks of age when myopathic facies and a head circumference of 36 cm (9-25th centile) were also recorded. By 2 months of age, limb movements were markedly decreased, and she was unable to fix and follow. Social smile was lost at 7 months. In addition, a clinical diagnosis of infantile spasms was supported by a hypsarrhythmic electroencephalogram (EEG) and anti-epileptic medications were commenced. Cranial magnetic resonance imaging (MRI) performed at 4 weeks and 7 months revealed diffuse parenchymal volume loss involving both grey and white matter (**Figure 1B**). The corpus callosum was hypoplastic with marked volume loss of cerebellar hemispheres and vermis at 7 months compared to 4 weeks. The mamillary bodies were absent and the hippocampi simplified with delayed myelination. S1 died aged 12 months due to her rapidly progressive neurodegenerative condition with refractory epilepsy.

Further clinical review by paediatric neurology led to a range of neurological, metabolic, genetic and radiological investigations that were diagnostically uninformative. Blood and fibroblasts were taken for cytogenetic (array CGH) and molecular genetic analysis.

S2, a male infant and younger sibling of S1, was born at 38+5 weeks gestation by normal vaginal delivery and weighed 3.615 kg (50th–75th centile) with a head circumference of 35.4 cm (25th-50th centile). No resuscitation was required at birth, but hypotonia and paucity of movement was noted. He had bilateral positional talipes with overlapping 4th and 5th toes on the right foot. Tachypnoea with low oxygen saturation prompted admission to the neonatal

intensive care unit where he was initially treated for transient tachypnoea of the newborn. A persistent oxygen requirement and feeding difficulties prompted further medical investigations.

At 2 weeks of age, recurrent seizures were treated with levetiracetam. Neurological examination revealed nystagmus, reduced anti-gravity movements and hypotonia. A cranial MRI at 11 days of age demonstrated a structural malformation with deep sulcation of the right occipital lobe and generalised (particularly vermian) cerebellar and corpus callosal hypoplasia. A comparison of S1 and S2 scans suggested a number of common features, most notably the vermian volume loss and callosal hypoplasia.

Ongoing feeding difficulties prompted insertion of a gastrostomy at 4 months of age. Around this time increased seizure activity was noted and while initial EEGs had been unremarkable, by 4 months the EEG was consistent with epileptic encephalopathy. Blood and fibroblasts were taken for cytogenetic (array CGH) and molecular genetic analysis. S2 died at 6 months of age following a presumed viral infection and subsequent increase in apnoeic episodes. The cause of death was recorded as a neurodegenerative condition with refractory epilepsy as in the case of S1, the older sibling.

Family 2

S3, a male, is the fourth child of healthy consanguineous, first cousin parents originating from the Middle East (Family 2, **Figure 1A**). His two oldest siblings are healthy. One older sister had difficulty with walking and speaking, impaired vision with nystagmus and died at the age of 7 years from end-stage kidney disease (ESKD). Many of these features were similar to S3, suggesting she may have had the same disease as S3; however no genetic analyses were performed. His mother also reported three previous pregnancy losses.

Pregnancy, term delivery and the neonatal period of S3 were normal. Limited detail is available on his early life, but psychomotor delay became evident during the first year and by 3 years he had experienced a single seizure though did not go on to develop epilepsy.

At 4 years of age, he was diagnosed with kidney disease, anaemia and thrombocytopenia. A clearly defined renal diagnosis has never been applied, but he subsequently developed ESKD. He received peritoneal dialysis from 8 until the age of 13 years when a renal transplant was performed. Renal ultrasound and renal MRI before the transplant showed bilateral atrophic kidneys with small cysts.

At 9 years of age, he was diagnosed with pancreatitis and cholecystitis, subsequently having a cholecystectomy. At 12 years of age, he also had surgical repair for large bilateral inguinal hernias. He was severely growth retarded with Z-score for length at -6 SD before the renal transplant. Growth remained impaired and did not improve substantially after transplantation. He has been diagnosed with hypothyroidism and receives thyroxine replacement therapy.

He has limited verbal language skills, moderate intellectual disability and wears hearing aids to assist with bilateral sensorineural hearing impairment. He walks only with the aid of a walker due to ataxia. He has had dry and scaly skin since early childhood. Brain MRI obtained at 12 years of age showed atrophy of both cerebellar hemispheres and the vermis and widespread bilateral polymicrogyria (**Figure 1B)**. An ophthalmological examination revealed bilateral optic atrophy and typical Bull's eye maculopathy.

His clinical appearance in adolescence is one of short stature, with a slender build. He has microcephaly, down slanting almond-shaped eyes and blepharophimosis. He has a broad nasal bridge, anteverted nostrils, long philtrum and cupid's bow upper lip. His ears are low set with an irregular helix, and he has sparse scalp hair (**Figure 1C**). He also has bilateral fifth finger clinodactyly of both hands.

Family 3

S4 is the first child of consanguineous parents of Indian heritage from the Daman region, India (Family 3, **Figure 1A-C**). He was born by lower segment cesarean section (birth weight of 2.88kg and HC of 34 cm) following a pregnancy complicated by gestational diabetes mellitus. An antenatal diagnosis of dilated urinary bladder was not confirmed on postnatal abdominal ultrasound scan, which was reported normal. Parents believed that he was well until concerns were raised at a six-week check by the health visitor who felt that he was not fixing and following. At about 3 months old he experienced his first seizure, which lasted for more than 45 minutes. Currently, he is on levetiracetam, vigabatrin and gabapentin along with the ketogenic diet. S4 has a younger brother who is now 3.5 months old and is well.

Family 4

S5 is a male child of healthy non-consanguineous parents (Family 4, **Figure 1A, C**). The proband's 6-year-old brother died of atypical brainstem glioma. The proband has one sister and she is well.

Pregnancy with S5 followed a natural conception and proceeded without incident until a fetal ultrasound scan at approximately 20 weeks gestation revealed talipes and dilated ventricles. The pregnancy was then monitored and S5 was born at 39 weeks gestation with a normal birth weight (3095g). His birth length was 47 cm and birth head circumference was 33cm. He was noted to have neonatal jaundice, poor feeding and tongue tie. He received phototherapy and was discharged home after 3 days. He was readmitted soon afterwards with abnormal movements. Although EEG was reported normal, he went on to have clinical seizures and brain MRI scan revealed dysgenesis of the corpus callosum, prominent cavum septum pellucidum, abnormal cerebellar vermis, small cerebellar hemispheres and a small posterior fossa (**Figure 1B**). In view of the above features, microarray analysis was requested which did not find any copy number variation. Rapid WES was then requested under R14 category as per national genomic test directory and the result was normal. Metabolic investigations including blood lactate, organic acids, amino acids, carnitine, very long chain fatty acids were normal. The child subsequently exhibited central hypotonia, microcephaly, significant global developmental delay, epileptic encephalopathy, cerebral irritability and movement disorder with dystonia and dyskinesia. Whole genome sequencing (WGS) was requested as a trio analysis with R27, R59, R57, R98, R69 panels as per national genomic test directory and the result was also normal.

Clinical review at 4 years 6 months showed significant hypotonia, profound developmental delay with a non-verbal and non-mobile child who has a gastrostomy *in situ*, intractable epilepsy and a movement disorder. Palliative care has been involved.

Family 5

S6 is male and the third child of consanguineous Pakistani parents. He presented at 7 months of age with concerns regarding developmental delay, failure to thrive and seizures from 1 month of age. Seizures were controlled by one year of age with levetiracetam 40mg/kg/day. Antenatal and post-natal history were not significant. He was born at term via spontaneous vaginal delivery. He was partially vaccinated and had multiple hospital admissions with uncontrolled seizures. An older brother had impaired vision with borderline intellectual disability, while an older sister had a history of urethral meatal stenosis but no developmental delay. A younger sister had multiple café au lait macules, global developmental delay, behavioural issues (aggression, hyperactivity) and recurrent chest and gastrointestinal infections.

On examination at age 7 months, occipitofrontal circumference (OFC) was 37.5cm, weight 4.5kg with clinical signs of malnutrition but no obvious dysmorphic features. He was floppy with poor truncal control, head lag and hyporeflexia. He appeared unable to grasp objects placed in his palms. He could fix on familiar faces but was unable to follow. Eye examination revealed vernal keratoconjunctivitis with bilateral pseudogerontoxon. Right eye had giant and limbal papillae while upper tarsal micropapillae were observed in left eye. Over time, he developed spasticity with failure to gain weight. Speech did not progress beyond simple vocalisations. EEG at 7 months of life was normal but no neuroimaging was obtained. At 2.5 years he was emaciated, weighing only 2kg (<0.4th centile), and passed away following a period of vomiting leading to severe dehydration.

Supplemental Figures

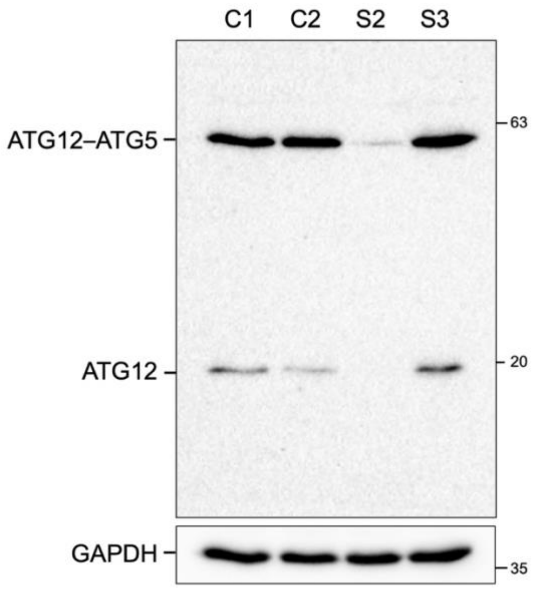

Figure S1. Full length western blot analysis of ATG12 species. Western blot analysis of patient derived fibroblasts produces two distinct bands corresponding to the ATG12-ATG5 heterodimer, and unconjugated ATG12. The c.363+3A>T *ATG12* variant observed in S2 does not generate any detectable additional ATG12 species.

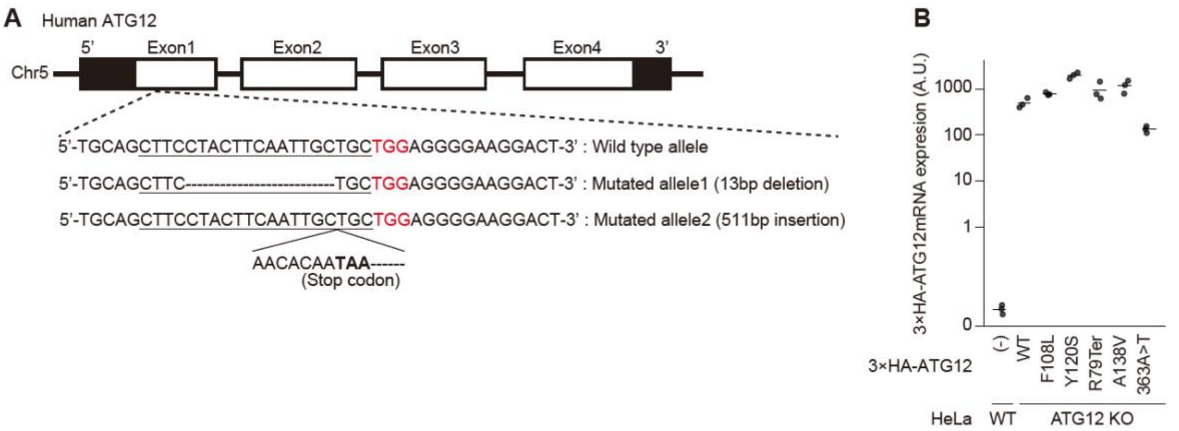

Figure S2. Generation of ATG12 knockout cells and *ATG12* mRNA expression of ATG12 variants. (**A**) Generation of ATG12 knockout (KO) cells. Schematic representation of the Cas9-gRNA-target site (underlined) in the *ATG12* genomic locus. The protospacer-adjacent motif sequence is shown in red. A deletion of 13 bp and an insertion of 511 bp in the knockout allele are indicated. **(B)** Expression levels of *ATG12* variants. The mRNA expression levels of each *ATG12* variant in control (HeLa WT) cells and ATG12-KO HeLa cells expressing the indicated constructs were quantified by qPCR. Transcripts derived from the 3×HA-tagged ATG12 constructs were detected using primers specific for the 3×HA tag (forward, TTCCTGACTATGCGGGCTAT; reverse, CAGCGTAATCTGGAACGTCATA). Relative expression levels were calculated using the ΔΔCt method and normalized to ActB, detected with the following primers (forward, CCTTCTTGGGTATGGAATCCTGT; reverse, CACTGTGTTGGCATAGAGGTCTTTAC).

Table S1. Primers used for genomic DNA confirmation of *ATG12* variants

Target	Primer sequence
ATG12_ex3F	GAAAGTCATCTTTTCTACCCCAGA
ATG12_ex3R	TTGTGGGTAAGTGGGGACAT
ATG12_Family2 F	ACAAGTAGGAGCAATGGGTGT
ATG12_Family 2 R	AGCTCATTCATGCCTAAGATGCT

Table S2. Primers used for cDNA investigations into *ATG12* splice variant.

Target	Primer sequence
ATG12_cDNA_F	GGATGTCTCCCCAGAAACAA
ATG12_cDNA_R	AACAAGTAGGAGCAATGGGTGT

Table S3. Genotype and source of *S. cerevisiae* used in this study.

Strain	Genotype	Source	Purpose
JH914	SEY6210+*atg12Δ::KANMX6 PGK1-GFP::natMX6*	This study	Pgk1-GFP assay
JH947	WLY176 *pRS405-ATG8p-GFP-ATG8::LEU2 atg12Δ::HIS3MX6 pRS406-ATG12p-3xHA-ATG12 F154L::URA3*	This study	Atg8 lipidation, GFP-Atg8, and Pho8Δ60 assays
JH948	WLY176 *pRS405-ATG8p-GFP-ATG8::LEU2 atg12Δ::HIS3MX6 pRS406-ATG12p-3xHA-ATG12 W166S::URA3*	This study	Atg8 lipidation, GFP-Atg8, and Pho8Δ60 assays
JH949	SEY6210+*atg12Δ::KANMX6 PGK1-GFP::natMX6 pRS406::URA3*	This study	Pgk1-GFP assay
JH950	SEY6210+*atg12Δ::KANMX6 PGK1-GFP::natMX6 pRS406-ATG12p-3xHA-ATG12::URA3*	This study	Pgk1-GFP assay
JH952	SEY6210+*atg12Δ::KANMX6 PGK1-GFP::natMX6 pRS406-ATG12p-3xHA-ATG12 F154L::URA3*	This study	Pgk1-GFP assay
JH953	SEY6210+*atg12Δ::KANMX6 PGK1-GFP::natMX6 pRS406-ATG12p-3xHA-ATG12 W166S::URA3*	This study	Pgk1-GFP assay
JH954	WLY176 *pRS405-ATG8p-GFP-ATG8::LEU2 atg12Δ::HIS3MX6 pRS406-ATG12p-3xHA-ATG12 A184V::URA3*	This study	Atg8 lipidation, GFP-Atg8, and Pho8Δ60 assays
JH955	SEY6210+*atg12Δ::KANMX6 PGK1-GFP::natMX6 pRS406-ATG12p-3xHA-ATG12 A184V::URA3*	This study	Pgk1-GFP assay
OC751	BY4742, *atg12::KanMX4, ura3Δ0, his3Δ1, leu2Δ0, lys2Δ0*	YKO collection	GFP-Atg8 *in-vivo* microscopy
OC588	BY4742, *atg8::GFP-ATG8-URA3, ura3Δ0, his3Δ1, leu2Δ0, lys2Δ0*	This study	GFP-Atg8 *in-vivo* microscopy
OC744	BY4742, *atg8::GFP-ATG8-URA3, atg12::KanMX4, ura3Δ0, his3Δ1, leu2Δ0, lys2Δ0*	This study	GFP-Atg8 *in-vivo* microscopy
YLY290	WLY176 *pRS405-ATG8p-GFP-ATG8::LEU2 atg12Δ::HIS3MX6 pRS406::URA3*	This study	Atg8 lipidation, GFP-Atg8, and Pho8Δ60 assays
YLY291	WLY176 *pRS405-ATG8p-GFP-ATG8::LEU2 atg12Δ::HIS3MX6 pRS406-ATG12p-3xHA-ATG12::URA3*	This study	Atg8 lipidation, GFP-Atg8, and Pho8Δ60 assays

Table S4. Oligonucleotides used to generate plasmid constructs for yeast complementation studies.

Target	Primer sequence
ATG12 Cloning	5’ AGTCTAGAACCTAGTCTTCCATTTACATCG 3’
	5’ AACTGCAGGGGTCTTTGGTGAAAAAGGG 3’
ATG12 W166S mutagenesis	5’ TTGGTGAACTTTCGATGCAATTCAAGAC 3’
	5’ TATTTTGCTGCGGACTTGGC 3’

ATG12 A184V	5’ TTGTGCATCCGTAGTGTTTGGTTAAATAC 3’
	5’ TAACTTACAATAAGCTCATCATTAGTC 3’

Table S5. Overview of phenotypes associated with described congenital disorders in *ATG* genes.

**Gene**	***ATG12***						***ATG7***	***ATG5***
**No. of individuals**	***n* = 6**						***n* = 12**	***n* = 2**
**Reference**	**Lambton *et al.* present study**						**Collier *et al* 2021**[8]	**Yapici *et al* 2005**[10] **Kim *et al* 2016**[9]
Subject	S1	S2	S3	S4	S5	S6	S1-12	Subject 1 and 2[a]
Age at last examination/ death	Died 12 m	4 m/died 6 m	17 y	6 y	4 y 6 m / died 5 y	7 m / died 2 y 6 m	(1-71 y)	(13 y and 15 y)
Ataxia	NA	NA	+ walks with support	Nonambulant	NA	Did not achieve walking	9/11	2/2
DD	+	+	+	+	+	+	12/12	2/2
Intellectual disability	+ EE	+ EE	+	+	+ EE	+	12/12	2/2
Seizures	+ IS	+	+	+ IS	+	+	2/12	0/2
CC hypoplasia	+	+	-	+	+(Body of cc thin and absent posteriorly)	N/A	7/9	0/2
Cerebellar (vermian) hypoplasia	+	+	+	+	+	N/A	7/11	2/2
Other brain anomaly	Reduced white and grey matter	Deep sulcation occipital lobe	Polymicrogyria (widespread)	Microcephaly with brachycephaly & cerebellocerebral atrophy, thinning of pons, brainstem. hypomyelination	Prominent cavum septum pellucidum, small cerebellar hemispheres, small posterior fossa	N/A	Brain volume loss 3/9	-

Sensorineural deafness	NA	NA	+	NA	NA	N/A	2/12	NA
Optic atrophy	NA	-	+	+	NA	No but vernal keratoconjunctivitis with bilateral pseudogerontoxon	7/9	NA
Kidney disease	NA	NA	ESKD	NA	-	-	NA	NA
Dysmorphology	NA	NA	+	+	+	-	4/12	NA
Growth retardation	NA	NA	+	+	+	+	3/8	NA
Other			Dry scaly skin, sparse hair, pancreatitis, cholecystitis, inguinal hernia, hypothyreosis		Movement disorder with dystonia and dyskinesia, cerebral irritability, PEG fed	Repeated chest and GI infections, failure to thrive, microcephaly		Club feet 1/2

+ = present; - = absent; CC = corpus callosum; CI = cognitive impairment; DD= developmental delay; EE = epileptic encephalopathy; ESKD = end-stage kidney disease; IS = infantile spasms; m = months, NA = not assessed; S = subject; y = years; PEG = percutaneous endoscopic gastrostomy. [a] Subject 1 and Subject 2 from family 1 in Yapici *et al* 2005; reassessed as subjects 601 and 602 in Kim *et al* 2016.

Further acknowledgments

We are grateful to the families for participating to the study. We thank Dr. Siren Berland and co-workers at Haukeland Universitetssjukehus, Bergen, Norway, for performing genetic testing in a clinical setting. We thank the staff of the HiLIFE electron microscopy unit (directed by Dr. Eija Jokitalo) for their excellent technical assistance, and Dr. Helena Vihinen for insightful advice on sample preparation. We are grateful to Professor Eeva-Liisa Eskilinen (University of Turku, FI) for insightful discussions and guidance on discriminating autophagic sub-compartments. We thank Yongheng Liang (Nanjing Agricultural University) for plasmid *pP1kGFP-ATG8(406)* and Florence Piron Prunier for technical advice. We also thank UNINETT Sigma2, the National Infrastructure for High Performance Computing and Data Storage in Norway, for support to store and analyse High-Throughput Sequencing data.
