# Supplementary material for: Bi-allelic ATG12 variants impair autophagy and cause a neurodevelopmental disorder
Source: Am J Hum Genet. 2026 Mar 26;113(5):1090–107. doi: 10.1016/j.ajhg.2026.03.002 (PMC13277686; doi:10.1016/j.ajhg.2026.03.002)
Supplement: Document S2. Article plus supplemental information [file mmc2.pdf]

Bi-allelic *ATG12* variants impair autophagy and cause a neurodevelopmental disorder

Graphical abstract

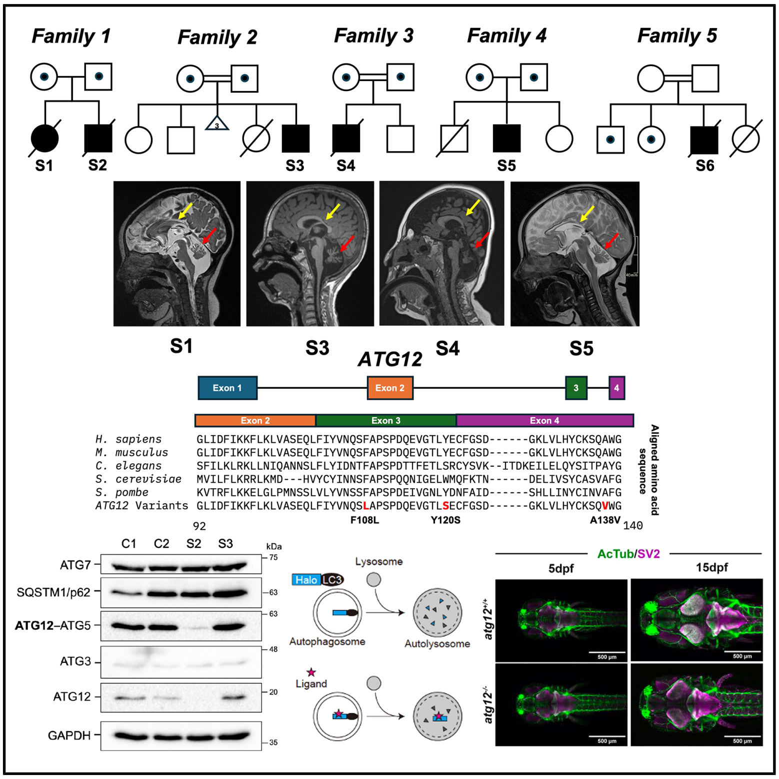

Authors

James Lambton, Shotaro Asano, Yuxiang Huang, ..., Monika Oláhová, Charlotte L. Alston, Robert W. Taylor

Correspondence

robert.taylor@ncl.ac.uk

We describe six individuals from five unrelated families harboring variants in *ATG12*, a core autophagy gene, resulting in a neurodevelopmental disorder. Using patient tissue and *in vitro* and *in vivo* models, we show that these variants disrupt autophagy, leading to neurological phenotypes. This work highlights the growing importance of congenital autophagy disorders.

Lambton et al., 2026, The American Journal of Human Genetics *113*, 1090–1107
May 7, 2026 © 2026 The Author(s). Published by Elsevier Inc. on behalf of American Society of Human Genetics.
https://doi.org/10.1016/j.ajhg.2026.03.002

Bi-allelic *ATG12* variants impair autophagy and cause a neurodevelopmental disorder

James Lambton,[1] Shotaro Asano,[2,3] Yuxiang Huang,[4] Fumi Suomi,[5,6] Tomoya Eguchi,[2] Cassidy Petree,[7] Kevin Huang,[7] Magali Prigent,[8,9] Aliza Imam,[10] Thomas J. McCorvie,[11] Daniel Warren,[12] Emma Hobson,[10] Helen McCullagh,[13] Doriana Misceo,[14] Anna Bjerre,[15] Marie F. Smeland,[16,17] Claus Klingenberg,[17,18] Eirik Frengen,[14] Swati Naik,[19] Gavin Ryan,[19] Annapurna Sudarsanam,[20] Katherine Foster,[21] Pradeep Vasudevan,[22] Rajib Samanta,[23] Fatima Rahman,[24] Shazia Maqbool,[24] Vrajesh Udani,[25,26] Stephanie Efthymiou,[27] Henry Houlden,[27] Robert McFarland,[1,28] Jack J. Collier,[29] Reza Maroofian,[27] Wyatt W. Yue,[11] Gaurav K. Varshney,[7] Daniel J. Klionsky,[3] Renaud Legouis,[7,8] Thomas G. McWilliams,[4,5] Noboru Mizushima,[2,30] Monika Oláhová,[1,31] Charlotte L. Alston,[1,28] and Robert W. Taylor[1,28,*]

Summary

Autophagy is an essential developmental and homeostatic process, defined by the endolysosomal degradation of intracellular components and pathogens. Dysfunctional autophagy is implicated in complex human disease, yet reports of congenital autophagy disorders were considered exceedingly rare until the recent report of several unrelated families with bi-allelic variants in the core autophagy effector *ATG7*, complementing the report of two individuals harboring *ATG5* variants. We now report six affected individuals from five families with bi-allelic *ATG12* variants with complex neurological phenotypes overlapping those seen in individuals with pathogenic variants in *ATG5* and *ATG7*: developmental delay, intellectual disability, congenital ataxia, hypotonia, and seizures with cerebellar vermis hypoplasia evident on neuroradiological imaging. Structural modeling implicated a potential disruption of the ATG12-ATG5-ATG16N-ATG3 complex. Biochemical analyses of primary fibroblasts confirmed the loss of stable ATG12-ATG5 conjugate in one family and altered autophagic flux in one unrelated family. The HaloTag processing assay in HeLa cells demonstrated a decrease in ATG12-ATG5 conjugate and reduced autophagic flux in response to starvation. Complementation studies demonstrated that equivalent missense *atg12* variants were unable to fully recover the biochemical defect in *atg12*-null yeast, with microscopy analysis demonstrating a reduced delivery of autophagy substrates to the yeast's degradative compartment. Zebrafish studies confirmed that Atg12 is required for normal growth, brain development, and neural function. Collectively, our findings underscore the pivotal role of autophagy in maintaining human neural integrity, emphasize an emerging group of congenital autophagy disorders, and expand our understanding of adaptive homeostasis in human health and disease.

[1]Mitochondrial Research Group, Translational and Clinical Research Institute, Faculty of Medical Sciences, Newcastle University, Newcastle upon Tyne NE2 4HH, UK; [2]Department of Biochemistry and Molecular Biology, Graduate School of Medicine, The University of Tokyo, Tokyo 113-0033, Japan; [3]Department of Ophthalmology, Graduate School of Medicine, The University of Tokyo, Bunkyo-ku, Tokyo, Japan; [4]Department of Molecular, Cellular and Developmental Biology, and Life Sciences Institute, University of Michigan, Ann Arbor, MI, USA; [5]Translational Stem Cell Biology & Metabolism Program, Research Programs Unit, University of Helsinki, 00290 Helsinki, Finland; [6]Department of Anatomy, Faculty of Medicine, University of Helsinki, 00290 Helsinki, Finland; [7]Genes & Human Disease Research Program, Oklahoma Medical Research Foundation, Oklahoma City, OK 73104, USA; [8]Institute for Integrative Biology of the Cell (I2BC), Université Paris-Saclay, CEA, CNRS, 91198 Gif-sur-Yvette, France; [9]INSERM U1280, 91198 Gif-sur-Yvette, France; [10]Leeds Clinical Genetics Service, Chapel Allerton Hospital, Leeds Teaching Hospitals NHS Trust, Leeds LS7 4SA, UK; [11]Biosciences Institute, Faculty of Medical Sciences, Newcastle University, Newcastle upon Tyne NE2 4HH, UK; [12]Department of Radiology, Leeds Teaching Hospitals NHS Trust, Leeds LS1 3EX, UK; [13]Department of Paediatric Neurology, Leeds Teaching Hospitals NHS Trust, Leeds LS1 3EX, UK; [14]Department of Medical Genetics, Oslo University Hospital and University of Oslo, 0450 Oslo, Norway; [15]Department for Specialised Paediatrics, Oslo University Hospital, 0450 Oslo, Norway; [16]Department of Paediatric Rehabilitation, University Hospital of North Norway, 9019 Tromsø, Norway; [17]Research Group for Child and Adolescent Health, Faculty of Health Sciences, UiT The Arctic University of Norway, Tromsø, Norway; [18]Department of Paediatrics and Adolescence Medicine, University Hospital of North Norway, Tromsø, Norway; [19]West Midlands Clinical Genetics Unit, Birmingham Women's and Children's Hospital, Birmingham B15 2TG, UK; [20]Department of Neurology, Birmingham Women's and Children's Hospital, Birmingham B15 2TG, UK; [21]Department of Radiology, Birmingham Children's Hospital, Birmingham B4 6NH, UK; [22]Department of Clinical Genetics, University Hospitals Leicester NHS Trust, Leicester Royal Infirmary, Leicester LE1 5WW, UK; [23]Department of Paediatric Neurology, University Hospitals Leicester NHS Trust, Leicester Royal Infirmary, Leicester LE1 5WW, UK; [24]Department of Developmental-Behavioral Pediatrics, The Children's Hospital and University of Child Health Sciences, Lahore, Pakistan; [25]P.D. Hinduja National Hospital and Medical Research Centre, Veer Savarkar Marg, Mahim, Mumbai 400 016, India; [26]Bai Jerbai Wadia Hospital for Children, Acharya Donde Marg, Parel, Mumbai 400012, India; [27]Department of Neuromuscular Diseases, UCL Queen Square Institute of Neurology, London, UK; [28]NHS Highly Specialised Rare Mitochondrial Disorders Service, Newcastle upon Tyne Hospitals NHS Foundation Trust, Newcastle upon Tyne NE1 4LP, UK; [29]Department of Clinical Neuroscience, University of Cambridge, Cambridge Biomedical Campus, Cambridge CB2 0QQ, UK; [30]International Research Center for Neurointelligence (WPI-IRCN), UTIAS, The University of Tokyo, Tokyo, Japan; [31]School of Geography and Natural Sciences, Faculty of Science and Environment, Northumbria University, Newcastle upon Tyne NE1 8SG, UK

*Correspondence: robert.taylor@ncl.ac.uk

https://doi.org/10.1016/j.ajhg.2026.03.002.

© 2026 The Author(s). Published by Elsevier Inc. on behalf of American Society of Human Genetics.
This is an open access article under the CC BY license (http://creativecommons.org/licenses/by/4.0/).

Introduction

Autophagy is a cellular degradation and recycling process that sustains eukaryotic homeostasis. During autophagy, cellular cargo is captured within double-membrane-bound vesicles known as autophagosomes, which fuse with acidic endolysosomes to form degradative autolysosomes.[1] At the molecular level, a highly conserved set of core autophagy-related (*ATG*) genes encode proteins that govern phagophore biogenesis, their maturation to autophagosomes, and their subsequent degradation by endolysosomes or vacuole. Briefly, upstream triggers are sensed and integrated by the unc-51-like autophagy-activating kinase 1 (ULK1) complex initiating phagophore formation via the PIK3C3/VPS34-containing class III phosphatidylinositol 3-kinase.[2] The E1-like enzymatic activity of homodimeric ATG7 aids the expansion of phagophore membranes via two ubiquitin-like conjugation systems. One of these systems involves ATG12, a ubiquitin-like protein that functions within a conjugation cascade to promote autophagosome formation.[3] ATG7-mediated adenylation of ATG12 results in its transfer to ATG5 via the E2-like ATG10, generating the ATG12-ATG5 conjugate at the phagophore membrane. ATG12-ATG5 forms a complex with ATG16L1 that facilitates mammalian Atg8-family protein (ATG8) lipidation, a covalent linkage with phosphatidylethanolamine (PE), allowing insertion into the phagophore membranes and subsequent autophagy.[4,5]

Extensive research over the last two decades has unveiled impairments in different aspects of autophagy across a spectrum of human diseases, spanning various cancers, neurodegenerative conditions, and kidney disorders.[6,7] Previously, we employed gene-agnostic whole-exome sequencing (WES) strategies that identified multiple unrelated families with bi-allelic pathogenic variants in *ATG7* (MIM: 608760), encoding a core autophagy effector.[8] Additionally, a pathogenic homozygous missense variant was previously reported in *ATG5* (MIM: 604261) in two siblings.[9,10] Cerebellar ataxia, cognitive impairment, and delayed motor milestones were consistently observed, emphasizing the importance of autophagy for human neural integrity (MIM: 619422 and 617584).[8–10] This is consistent with pre-clinical and developmental studies, where conditional ablation of *Atg5* or *Atg7* in the mouse nervous system leads to neuropathology.[11,12] Whole-body mutants of core *Atg* genes, including *Atg12*, results in highly penetrant perinatal lethality in mice, which might suggest that bi-allelic loss-of-function *ATG12* variants would be incompatible with human life.[13–15]

Here, we report the identification of six affected individuals from five unrelated families with bi-allelic variants in *ATG12* (MIM: 609608). These children presented with neurodevelopmental and neurological deficits, including hypoplasia of the cerebellar vermis, infantile hypotonia, ataxia, seizures, developmental delay, and/or intellectual disability. *In silico* and experimental analyses using primary fibroblast cells, HeLa cell models, yeast models, and zebrafish models illustrate the deleterious impact of these variants, underscoring the critical importance of autophagy in human neural development and disease.

Material and methods

Ethics and consent

Individuals and their families have consented to reporting of their data according to the Declaration of Helsinki. Ethical approval was obtained from all centers: Family 1 was identified as part of a National Institute of Health Research (NIHR) funded trio WES project ("Diagnosis of Mitochondrial Disease without Muscle"; IRAS 255610; REC: 20/NE/0272); family 2 was identified through a study approved by the Regional Committee for Medical Research Ethics – South-East Norway, REK 2010/1152a; families 3 and 4 were identified using database look-up as part of NIHR-funded trio WES project ("Diagnosis of Mitochondrial Disease without Muscle"; IRAS 255610; REC: 20/NE/0272); the investigation of family 5 is covered by UCL IRAS project ID: 310045. The families and associated researchers were connected through the GeneMatcher platform.[16]

Genetic analysis

Whole-genome and whole-exome sequencing

All subjects underwent genetic investigations using EDTA blood-derived DNA. For family 1 (S1 and S2), gene-agnostic whole-genome sequencing (WGS) of S1 and her parents was performed via the National Health Service (NHS) testing pathway (R14: acutely unwell children with a likely monogenic disorder). Subsequently, S2 and his parents were recruited to a NIHR-funded trio WES project for research-based analysis. Concurrent singleton WES was performed for S1 to allow comprehensive data analysis to be performed as a quad, with both affected children being analyzed alongside both unaffected parents. EDTA blood DNA from S1, S2, and their parents underwent library preparation using the Twist Exome 2.0 reagent (Twist Biosciences) and sequencing on an Illumina NovaSeq 6000 sequencer using 2×150-bp paired-end reads. Reads were aligned to the GRCh38 human genome build using the Burrows-Wheeler Aligner (BWA)[17]; variant calling was performed using the GATK HaplotypeCaller (version 3.4). All variants were annotated using the Ensembl Variant Effect Predictor (VEP), and a minor allele frequency (MAF) <0.01 (gnomAD v3.1) was used to define the rare-variant dataset for each member of the quad for family 1.[18] Each variant in S1 and S2 was first denoted as maternal or paternal, then genes with bi-allelic variants segregating between the two siblings were determined. SpliceAI was used to predict the likely impact of putative splicing variant.

For family 2 (S3), singleton WES was performed. FASTQ files were aligned to the GRCh38 human-genome reference sequence using BWA v.0.7.8. with duplicate reads removed using Picard v.1.119 (http://broadinstitute.github.io/picard/); indel realignment, base-quality recalibration, and variant calling were performed using Genome Analysis Toolkit (GATK) v.4.2.4.1.[17,19,20] Variant annotation was performed using the Ensembl VEP.[18] The variant calling file (VCF) was analyzed using the FILTUS program.[21] Variants with allelic frequency > 0.01 in gnomAD v3.1.2 (gnomad.

broadinstitute.org) or with a combined annotation-dependent depletion (CADD) PHRED score < 15 *in silico* tool were discarded.[22,23] Finally, prioritization of variants focused on missense, nonsense, frameshift, and small insertion/deletion variants, with an anticipated autosomal recessive (homozygous and compound heterozygous) and autosomal dominant (*de novo*) mode of inheritance.

Variants were interpreted according to the American College of Medical Genetics and Genomics (ACMG) 2015 and 2020 and Association for Clinical Genomic Science (ACGS) 2024 guidelines.[24,25] The presence of all *ATG12* variants and their segregation across family members was undertaken using Sanger sequencing.

Sanger-sequencing confirmation of ATG12 variants

Blood-derived genomic DNA was subject to PCR amplification and Sanger sequencing to confirm the presence of the identified *ATG12* variants (Table S1). PCR products were purified using *exo*I and shrimp alkaline phosphatase (Promega) and Sanger sequenced using the ABI BigDye terminator cycle-sequencing kits v3.1 (Life Technologies, Carlsbad, CA) before capillary electrophoresis using a 3730xl DNA analyzer running proprietary DNA Sequencing Analysis Software v. 5.1 (Applied Biosystems, Foster City, CA). Sequences were visualized using FinchTV (family 1) and SeqScape Software v.2.7 (family 2) (Thermo Fisher Scientific).

RNA studies to investigate a putative splicing variant in ATG12

Complementary DNA (cDNA) was derived from whole RNA extracted from affected individuals and age-matched control fibroblasts cultured in standard Dulbecco's modified Eagle's medium (DMEM). Reverse transcription was performed using Moloney murine leukemia virus reverse transcriptase reverse transcription system and random hexamer primers (Promega). Primers designed to amplify cDNA spanning from exon 1 of *ATG12* through to its 3′ UTR were used for PCR amplification (Table S2). Electrophoretic separation of the PCR amplicons using a 2% agarose gel facilitated identification of normal- and abnormal-length cDNA fragments. Amplicons were subject to Sanger sequencing to determine the effect of the *ATG12* variant (c.363+3A>T) on mRNA splicing.

Protein modeling

The previously described crystal structure for the ATG12-ATG5-ATG16N-ATG3 complex was obtained from RCSB Protein Data Bank (PDB: 4NAW).[26] To generate a model of the full-length ATG12-ATG5-ATG16N-ATG3 complex, the canonical full-length sequences were obtained from Universal Protein Knowledgebase (UniProt: Q9H1Y0-1, O94817-1, Q9NT62-1, Q676U5-1).[27] The multimeric complex was then modeled using AlphaFold-MULTIMER using standard settings.[28]

Assessment of autophagy in primary cell lines

Immunoblotting analysis

Primary fibroblasts derived from study subjects were cultured in Gibco DMEM (4.5 g/L glucose, 2 mM L-glutamine, 1 mM pyruvate) with 10% fetal bovine serum (FBS), 50 μg/mL uridine, 1× non-essential amino acids, and 1× penicillin-streptomycin and incubated at 37°C in 5% CO_2 and lysed using cold lysis buffer (50 mM Tris/HCl pH 7.4, 130 mM NaCl, 2 mM $MgCl_2$, 1 mM PMSF, 1% Nonidet P-40, one cOmplete EDTA-free protease inhibitor tablet [Roche]). SDS-PAGE was performed using 8% or 12% polyacrylamide gels.

Membranes were blocked for 1 h at room temperature in 5% skimmed milk powder (Marvel) dissolved in Tris-buffered saline (Santa Cruz) with 0.1% Tween 20 (Sigma) (TBS-T). Immunoblotting was performed either overnight at 4°C or for 1 h at room temperature with primary antibodies. Immunoblotting was performed using the following antibodies: ATG12 1:1,000 (Abcam ab303488), ATG7 1:1,000 (Cell Signaling Technology #8558), ATG3 1:500 (Cell Signaling Technology #3414), ATG5 1:500 (Cell Signaling Technology #2630), SQSTM1/p62 1:1,000 (Abcam ab109012), MAP1LC3B/LC3B 1:1,000 (Cell Signaling Technology #2775), GAPDH 1:10,000 (Proteintech 60004-1-Ig), anti-rabbit 1:2,000 (DAKO P0399), and anti-mouse 1:2,000 (DAKO P0260). Following this, membranes were incubated in species-specific horseradish peroxidase (HRP)-conjugated secondary antibody for 1 h at room temperature. Membranes were visualized using SuperSignal West Pico PLUS Chemiluminescent Substrate (Thermo Scientific) and imaged with a ChemiDoc XRS+ Imaging system using Image Lab Software (Bio-Rad).

Autophagy flux assay

Primary proband-derived and age-matched control fibroblasts were seeded at 100,000 cells per 60-mm dish and cultured for 2–3 days as described to reach ~60% confluency. Subsequently, cells were treated with an unsaturating (2 nM) or saturating (100 nM) concentration of bafilomycin A_1 ($BafA_1$; Enzo Life Sciences) alongside basal conditions or starvation (Hanks' balanced salt solution [HBSS; Gibco]) conditions and at 37°C, 5% CO_2. Cells were harvested after 3 h for immunoblot analysis.

Bulk autophagic sequestration activity assay (LDH sequestration assay)

Autophagic sequestration assays were performed by measuring the activity of autophagosomal lactate dehydrogenase (LDH) as previously described.[29,30] Primary fibroblasts were cultured using standard tissue culture conditions, at 37°C, 5% CO_2 in DMEM with high glucose (Gibco), supplemented with 1× GlutaMAX, 10% FBS, and 100 U/mL penicillin-streptomycin. To induce autophagy, cultures were acutely deprived of serum and amino acids using EBSS with 100 nM $BafA_1$ for 3 h at 37°C, 5% CO_2. Cells were harvested using 0.25% trypsin-EDTA and collected in PBS containing 5% BSA. After centrifugation at 500 × *g* for 5 min at 4°C, cells were resuspended in sucrose solution (10% w:v, ice-cold). Subsequently, plasma-membrane disruption was accomplished using electroporation (Bio-Rad, 2 kV, 25 μF, and 400 Ω with a pulse duration of ~8 ms). Cells were resuspended in 400 μL of phosphate-buffered sucrose (100 mM sodium monophosphate, 2 mM EDTA, 2 mM DTT, 1.75% sucrose, pH 7.5). Following this, 550 μL of the disrupted cell suspension was resuspended in 900 μL of ice-cold resuspension buffer (50 mM sodium monophosphate, 1 mM EDTA, 1 mM DTT, pH 7.5) for the sedimented measurement ($LDH_{\text{“Sediment”}}$). Autophagic vacuoles were then sedimented by centrifugation at 18,000 × *g* for 45 min at 4°C. The resulting supernatant was aspirated and flash frozen using liquid nitrogen and stored at −80°C.

For total LDH measurement, 100 μL from the disrupted cell suspension solution was collected, flash frozen with liquid nitrogen, and stored at −80°C ($LDH_{\text{“Total”}}$). $LDH_{\text{“Sediment”}}$ was diluted in resuspension buffer with 1% Triton X-405 and $LD_{\text{“Total”}}$ with 1.5% Triton X-405 in a cold room for 30 min with agitation. Subsequently, samples were centrifuged at 18,000 × *g* for 5 min at 4°C, after which LDH activity was measured as described previously in a working solution containing 0.6 mM pyruvate and 0.36 mM NADH.[29,30]

Assessment of autophagy in HeLa *ATG12* knockout models

Cell culture

HeLa cells, which were authenticated by RIKEN, were used in this study. Cells were cultured in DMEM (Sigma-Aldrich) supplemented with 10% FBS (Sigma-Aldrich) at 37°C, 5% CO_2. For starvation, cells were treated with amino acid-free DMEM without FBS.

Reagents and antibodies

Anti-HA (Cell Signaling Technology #3724), anti-ATG12 (MLB M154-3), anti-HSP90 (BD Transduction Laboratories 610419), anti-LC3A/B (Cell Signaling Technology #12741), and anti-SQSTM1/p62 (PROGEN GP62-C) antibodies were used as primary antibodies. HRP-conjugated anti-mouse IgG (Jackson ImmunoResearch Laboratories 111-035-003), HRP-conjugated anti-rabbit IgG (Jackson ImmunoResearch Laboratories 111-035-144), and HRP-conjugated anti-guinea pig IgG (Jackson ImmunoResearch Laboratories 106-035-003) antibodies were used as secondary antibodies.

Generation of ATG12 *knockout HeLa cells*

An *ATG12* knockout (KO) HeLa cell line was generated using the CRISPR-Cas9 gene editing technique. A CRISPR guide RNA (gRNA) targeting *ATG12* (exon 1: 5′-CTTCCTACTTCAATTGCTGC-3′) was inserted into pSpCas9(BB)-2A-Puro (PX459) V2.0 plasmid (a gift from Feng Zhang; Addgene plasmid #62988; http://n2t.net/addgene:62988; RRID: Addgene_62988) and transfected into HeLa cells using Lipofectamine 2000 (Thermo Fisher Scientific) following the manufacturer's protocol.[31] Puromycin (Sigma) selection was started 1.5 days after transfection. KO clones were obtained by cell sorting using CytoFLEX SRT (Beckman Coulter Life Science).

Constructs and plasmids

The human *ATG12* cDNA (GenBank: NM_004707.4), inserted with intron 3, was cloned into pMRX-IPU with an N-terminal 3×HA tag. *ATG12* variants were generated by site-directed mutagenesis. *LC3* was inserted into pMRX-IBU-HaloTag7 to create pMRX-IBU-HaloTag7-LC3.

Retrovirus infection

HEK293T cells were transfected with retroviral plasmids together with packaging and envelope plasmids (pCG-gag-pol and pCG-VSVg) using Lipofectamine 2000 (Thermo Fisher Scientific), according to the manufacturer's protocol. Three days after the transfection, the retrovirus-containing medium was collected with a 0.45-μm-pore filter and transferred to the recipient cells. After 2 days, selection was performed with 2 μg/mL puromycin (Sigma), 2.5 μg/mL blasticidin (Fujifilm Wako Pure Chemical Corporation), or 50 μg/mL hygromycin (Nacalai Tesque).

Quantitative real-time PCR

HeLa cells were plated in six-well culture plates. Total cellular RNA was extracted using the RNeasy Plus Mini Kit (QIAGEN, 74134). cDNA was synthesized from equal amounts of RNA (50 ng/μL) using the ReverTra Ace qPCR RT Master Mix with gDNA Remover (TOYOBO, FSQ-301). Transcripts derived from the 3×HA-tagged ATG12 constructs were detected using primers specific for the 3×HA tag (forward, TTCCTGACTATGCGGGCTAT; reverse, CAG CGTAATCTGGAACGTCATA). Relative expression levels were calculated using the ΔΔCt method and normalized to ActB, which was detected with the following primers (forward; CCTTCTTGGGT ATGGAATCCTGT; reverse, CACTGTGTTGGCATAGAGGTCTT TAC). Real-time PCR was performed on a Thermal Cycler Dice Real Time System III (Takara Bio).

HaloTag processing assay

Cells were pulse-labeled with tetramethylrhodamine-conjugated ligand (100 nM) for 20 min, and the ligands were washed out. After starvation, cells were lysed in a lysis buffer (PBS containing 1% Triton X-100 and protease inhibitors; Nacalai Tesque) and incubated on ice for 30 min. The lysates were collected and centrifuged at 12,000 × *g* for 15 min. The supernatant was collected and separated by SDS-PAGE. The gel was analyzed with Odyssey (LI-COR) immediately after SDS-PAGE.[32]

Immunoblotting

Cell lysates were separated by SDS-PAGE and transferred onto polyvinylidene fluoride (PVDF) membranes. The membranes were blocked with 4% non-fat milk powder in Tris-buffered saline with 0.1% Tween 20 and incubated with primary antibodies at 4°C overnight. After washing, the membranes were incubated with species-appropriate secondary antibodies (1:10,000) at room temperature for 1 h. Signals were detected using FUSION Solo.7S.EDGE (Vilber Lourmat) with Immobilon Western Chemiluminescent HRP Substrate (Millipore).

Assessment of autophagy in *Saccharomyces cerevisiae Atg12* mutants

Yeast strains, media, and growth conditions

Yeast strains used in this study are listed in Table S3. Yeast cells were cultured at 30°C in rich medium (YPD; 1% yeast extract, 2% peptone, and 2% glucose). To induce autophagy, cells in the mid-log phase (optical density 600 [OD_{600}] = 0.8–1.0) were shifted to nitrogen-starvation medium with glucose (SD-N; 0.17% yeast nitrogen base without ammonium sulfate or amino acids, and 2% glucose) for the indicated times.

Autophagic flux assays and western blotting

Atg8-PE lipidation, GFP-Atg8 processing, Pho8Δ60 activity, and Pgk1-GFP processing assays were performed as previously described.[33–35] Antisera were from the following sources: Atg8,[36] Pgk1 (a generous gift from Dr. Jeremy Thorner, University of California, Berkeley), monoclonal YFP (Clontech, 632381), and monoclonal HA (Sigma, H3663). The blot was imaged using a ChemiDoc Touch imaging system (Bio-Rad) and quantified using Bio-Rad Image Lab software.

Yeast strains and growth media for fluorescence microscopy

S. cerevisiae strains used in this study are listed in Table S3. OC588 (BY4742, *atg8*::*GFP-ATG8-URA3*, *ura3Δ0*, *his3Δ1*, *leu2Δ0*, *lys2Δ0*) was obtained by integration of plasmid *pP$_{1K}$GFP-ATG8(406)* at the BY4742 chromosomal *ATG8* locus.[35] The *GFP-ATG8 atg12Δ* (OC744) strain was created by genomic integration of a deletion cassette PCR fragment amplified from the OC751 strain in the *GFP-ATG8* (OC588) strain.

Yeast cells were grown to log phase in complete synthetic medium (CSM: 0.17% yeast nitrogen base, 0.5% ammonium sulfate, 2% glucose, and amino acids without uracil or leucine). YNB-N (0.17% yeast nitrogen base without ammonium sulfate and amino acids and 2% glucose) was used as nitrogen-starvation medium.

ATG12 with 500-bp upstream and downstream regions was amplified by PCR, cloned into the TA pCR4-TOPO vector (Thermo Fisher Scientific), and sequence verified. Plasmids encoding Atg12^{W166S} or Atg12^{A184V} were generated using the Phusion Site-Directed Mutagenesis kit (Thermo Scientific) in pCR4-*ATG12*. Wild-type (WT) *ATG12* and mutant sequences were cloned into the pRS315 vector at *Xba*I and *Pst*I sites (Table S4).

Fluorescence microscopy

Cells were grown to log phase in CSM without uracil and leucine and then were transferred to nitrogen-starvation medium for 4 h. At different time points, cells were collected and observed with a three-dimensional deconvolution microscope DMIRE2 (Leica Microsystems) equipped with an HCxPL APO 100× oil CS objective, NA = 1.40 (Leica Microsystems) and an incubation chamber. Images were captured with a 20-MHz Cool SNAPHQ2 charge-coupled device camera (Roper Technologies) with a z-optical spacing of 0.2 μm. Metamorph software (Molecular Devices) was used to acquire z series and to deconvolve the images.

Statistical analysis

For the bulk autophagic sequestration assay, ordinary one-way analysis of variance (ANOVA) and Sidak's multiple comparison tests were performed to assess the differences between samples. Graphs and statistics for yeast data were performed using GraphPad Prism 8.0 software. The tests used Kruskal-Wallis or two-sided Fisher's exact test, and the statistical significance is indicated in the figure legends.

Developmental assessment of *atg12*-null zebrafish

Ethics statement and zebrafish husbandry

All procedures involving experimental animals were conducted in accordance with institutional policies and NIH guidelines. Zebrafish (*Danio rerio*) were housed and maintained under standard conditions in an AAALAC-accredited facility at the Oklahoma Medical Research Foundation (OMRF). All experimental protocols were reviewed and approved by the OMRF Institutional Animal Care and Use Committee (IACUC; protocol #22-76).

Generation of atg12 *KO zebrafish*

We used the CRISPR-Cas9 method to generate *Atg12* KO animals according to established protocols.[37,38] Briefly, a guide sequence was designed using the CRISPOR tool, and gRNA was chemically synthesized by Synthego (CA, USA). A 6-μL mixture containing 1 μL of 40 μM Cas9-NLS protein (University of California Berkeley QB3 Macrolab, Berkeley, CA, USA), 500 ng of gRNA (in 3 μL), and 2 μL of 1 M potassium chloride was injected into one-cell-stage embryos.[39] Founder embryos were raised to adulthood and outcrossed with WT to obtain heterozygous carriers, which were identified by genotyping the F1 progenies using fluorescent PCR.[38] Heterozygous carriers were subsequently inbred to generate homozygous animals for functional analysis, and indels were confirmed by Sanger sequencing.

Morphological phenotyping

Phenotyping was performed in homozygous animals by breeding two heterozygous carriers. Progeny from heterozygous crosses were monitored daily until 15 days post fertilization (dpf). The larvae were manually positioned in 2% methylcellulose (Sigma, USA) under a stereomicroscope for visualization and image capture. Bright-field images were captured using a Nikon DS-Fi2 high-definition camera mounted on a Nikon SMZ18 stereomicroscope (Nikon, Japan) equipped with auto-z stacking capability. After imaging, each embryo/larva was genotyped as described earlier.[38]

Whole-mount immunohistochemistry

Whole-mount immunohistochemistry was performed to label brain anatomical structures following established protocols.[40] The antibodies used are mouse anti-acetylated-tubulin antibody (1:500, Sigma-Aldrich T7451), rabbit anti-acetylated-tubulin antibody (1:250, Cell Signaling Technology #5335) and mouse anit-SV2A antibody (1:500, DSHB SV2). The secondary antibodies used are goat anti-mouse IgG Alexa Fluor 647 antibody and goat anti-rabbit IgG Alexa Fluor 488 antibody (1:500; Jackson ImmunoResearch Laboratories). Samples were mounted in 1.2% agarose and imaged using a light-sheet microscope.

Behavioral assay

All behavioral assays were performed at room temperature (RT) as previously described.[40,41] For the light/dark transition (LDT) test, 4-dpf larvae were individually placed in 96-well plates containing 150 μL of embryo water. At 5 dpf, the plate was transferred to a Noldus chamber, and locomotor activity was recorded using the DanioVision system with EthoVision XT software (Noldus Information Technology, Leesburg, VA, USA). Following a 30-min light-habituation period, larvae were subjected to two alternating cycles of 30 min in darkness and 30 min in light. Locomotor activity was quantified as distance traveled (mm/min), and minute-by-minute data were plotted using GraphPad Prism (GraphPad Software, San Diego, CA, USA).

Results

Genomic analyses reveal bi-allelic *ATG12* variants

We have investigated six affected individuals from five families with complex neurological phenotypes, characterized by developmental delay, intellectual disability, congenital ataxia, hypotonia, and seizures. The detailed clinical descriptions of all five families, including pedigrees and variant segregation data, brain MR imaging demonstrating posterior atrophy of the corpus callosum and cerebellar vermian hypoplasia, and clinical photography showing craniofacial abnormalities are presented in the supplemental information and Figures 1A–1C.

Microarray analysis (array CGH) undertaken for siblings S1 and S2 revealed an 11-kb copy number loss at 9p24.3 (including exons 40–44 of *DOCK8*; MIM: 611432) for S1 only; there was no phenotypic correlation, so this was not further investigated. Rapid, gene-agnostic trio WES analysis for S1 identified bi-allelic variants of uncertain significance in *SEMA6B* (MIM: 608873) and *PRMT7* (MIM: 610087); however, these were not found to segregate with S2. Repeat, research-based WES sequencing was undertaken for S1 and S2 and both parents considering their shared but undiagnosed presentation. Initial investigation of variants that segregated between both siblings and that were biparentally inherited failed to identify any suitable candidates, but, by focusing on variants that segregated across the two affected siblings (independent of parental data), two heterozygous variants were identified in *ATG12*. First, *ATG12*: c.359A>C (GenBank: NM_004707.4) (p.Tyr120Ser) missense variant with a CADD PHRED score of 24.4 and Rare Exome Variant Ensemble Learner (REVEL) score of 0.288. The variant has been reported on gnomAD v4.1.0 with 24 heterozygotes recorded, equating to an allele frequency of 2.578×10^{-5}. Subsequent analysis of the familial data revealed this variant to be paternally transmitted. Following completion of the functional experiments described in this manuscript, the *ATG12* c.359A>C (p.Tyr120Ser) variant is now classified as "likely pathogenic" according to the ACMG variant classification guidelines, fulfilling the following criteria: PS3_moderate

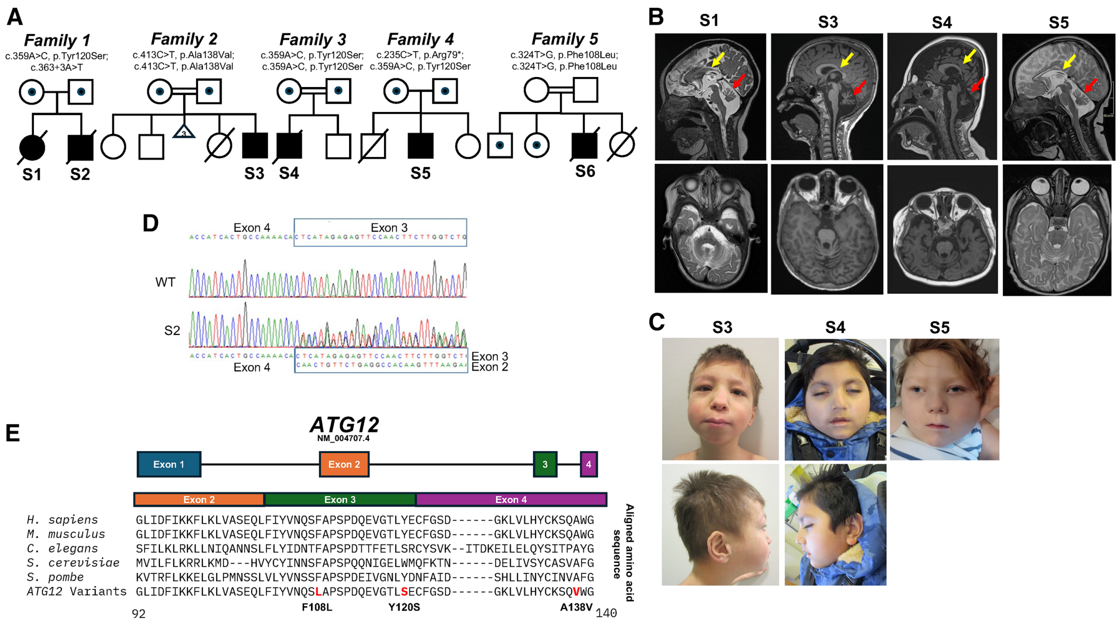

Figure 1. Family pedigrees and identification of *ATG12* variants
(A) Pedigrees of families 1–5 showing their *ATG12* genotypes.
(B) Brain MRIs of S1 at 7 months of age (T2 weighted), S3 at 12 years of age (T1 weighted), S4 at 12 years of age (T1 weighted), and S5 at 4 months of age (T2 weighted) showing posterior atrophy of the corpus callosum and cerebellar vermian hypoplasia, denoted by yellow and red arrows, respectively.
(C) Clinical photography of S3 at 12 years of age, S4 at 6 years of age, and S5 at 4 years of age, showing craniofacial abnormalities.
(D) Sanger sequencing of S2 *ATG12* cDNA showing splicing defects due to the c.363+3A>T variant. The double peaks detected are in line with the sequences of *ATG12* exon 2 and 3 as indicated.
(E) Schematic of ATG12 (RefSeq: NM_004707.4), with multiple sequence alignment of orthologs from various species, aligned using Clustal Omega, demonstrating the conservation of the amino acid residues involved.

(2 points), PM1_supporting (1 point), PM2_moderate (2 points), and PP4_supporting (1 point).[24,25] Additionally, an *ATG12*: c.363+3A>T (GenBank: NM_004707.4) putative splice variant was identified that was detected in both children but was not represented in the variant list for either parent. This variant has been reported in gnomAD v4.1.0 with 226 heterozygotes, equating to an allele frequency of 2.373×10^{-5} including one homozygote. The *ATG12* c.363+3A>T putative splice variant is now classified as likely pathogenic according to the ACMG variant classification guidelines, fulfilling the following criteria: PM1_supporting (1 point), PM3_moderate (2 points), PM4_moderate (2 points), and PP4_supporting (1 point). The variant has a CADD PHRED score of 24.2 and SpliceAI score of 0.830. Visualization of the BAM files using the Integrative Genomics Viewer (IGV) allowed visual confirmation of the maternal putative splice variant, and there is an adjacent polynucleotide tract that proffers an explanation as to why the maternal variant was not called.[24,25]

We performed WES of S3, and data analysis according to a recessive mode of inheritance identified the following homozygous missense variant ATG12: c.413C>T (GenBank: NM_004707.4) (p.Ala138Val), with a CADD PHRED score of 31 and REVEL score of 0.908. The variant is reported with an allele frequency of 1.12 × 10-5 in gnomAD v4.1.0, corresponding to 18 heterozygotes. Following completion of the functional experiments described in this manuscript, the ATG12 c.413C>T (p.Ala138Val) variant is now classified as likely pathogenic according to the ACMG variant classification guidelines, fulfilling the following criteria: PM1_supporting (1 point), PM2_moderate (2 points), PM3_supporting (1 point), PP3_supporting (1 point), and PP4_supporting (1 point).[24,25] Sanger sequencing of the family trio confirmed the variant to be homozygous in S3 and heterozygous in his healthy parents. No sample was available from the sister, possibly affected by the same disease. No genetic variants were identified that explained the kidney involvement reported in family 2, and a separate etiology is possible.

Having successfully used GeneMatcher to identify one additional individuals (S3), the detailed assessment of genomic data within the Genomic Medicine Service (GMS) Clinical Variant Ark (https://ip-cva-help.genomicsengland.co.uk/latest/)—a variant repository from individuals who have undergone WGS studies through the NHS GMS or the 100,000 Genomes Project (https://www.genomicsengland.co.uk/initiatives/100000-genomes-project)—revealed two additional families: S4, who was homozygous for the c.359A>C (p.Tyr.120Ser) *ATG12* variant; and S5, who also harbored a heterozygous c.359A>C (p.Tyr120Ser) *ATG12* variant in *trans* with a heterozygous c.235C>T (p.Arg79*) *ATG12* nonsense variant. This variant has been reported in gnomAD v4.1.0 with 58 heterozygotes, equating to an allele frequency of 3.595×10^{-5}.

WES of the proband of family 5 (S6) was performed as previously described.[42] Following the exclusion of pathogenic or likely pathogenic variants in currently known genes associated with neurological disorders, novel etiologies were interrogated and a homozygous missense variant in *ATG12*, located within a large region of homozygosity, was identified. Segregation analysis confirmed co-segregation of the homozygous c.324T>G (p.Phe108Leu) missense variant in *ATG12* with the disease phenotype. This variant has not been reported in gnomAD v4.1.0.

The c.363+3A>T *ATG12* variant causes aberrant mRNA splicing

Analysis of fibroblast-derived RNA from S2 demonstrated skipping of exon 3 (Figure 1D); this was predicted to cause an in-frame deletion of exon 3, consisting of 21 residues. The presence of a homozygous individual on gnomAD could suggest a hypomorphic mechanism, in that the c.363+3A>T variant causes "leaky" splicing, and there are sufficient residual WT transcripts to compensate; visual inspection of the IGV output from this individual shows some reads exhibit WT sequence, which would suggest that the gnomAD "homozygous" case may be mosaic or more likely represents a possible miscall due to the low complexity nature of the region. This is supported by the read-depth pileup across the wider region, where a range of different-size indels are visible at the 5′ end of the polyT tract, consistent with a region of low complexity.

Analysis of the splicing data also supports the mechanism relating to the paternal c.359A>C variant. Given the variant's proximity to the exon-intron boundary, it was postulated that this could cause aberrant splicing, but this was not supported by SpliceAI (score of 0.03, possible splicing significance is >0.2), or cDNA sequencing data. The c.359A>C variant is therefore likely to cause the predicted tyrosine-to-serine amino acid substitution (p.Tyr120Ser).

ATG12 variants might result in disrupted ATG12-ATG5-ATG16N-ATG3 interaction

The three missense variants identified (p.Phe108Leu, p.Tyr120Ser, and p.Ala138Val) map to the surface of the C-terminal domain of ATG12, which may imply roles in protein interactions (Figure 2A). The crystal structure of ATG12-ATG5, in complex with the N-terminal minimal interacting regions of ATG16L1 (ATG16N) and ATG3, has previously been determined to 2.2-Å resolution (Figure 2B).[26] Using AlphaFold-MULTIMER, we expanded the current ATG12-ATG5-ATG16N-ATG3 structure to include full-length ATG3 in the modeling.[28,43] Our expanded model recapitulates the reported interaction surface between ATG12 and ATG5 but reveals beyond the crystal structure how full-length ATG3 could wrap around ATG12 extensively (Figure 2C).

The three missense variants identified (p.Phe108Leu, p.Tyr120Ser, and p.Ala138Val) map to different segments of ATG12, all three of which lie close to ATG12's interfaces with ATG5 and ATG3. The Ala138 lies on the external face of ATG12, in very close proximity to ATG3, suggesting the ATG12 p.Ala138Val variant may interrupt ATG3 association with the ATG12-ATG5 complex. In contrast, the Phe108 residue lies directly adjacent to ATG5, suggesting the ATG12 p.Phe108Leu variant may affect ATG12-ATG5 heterodimer formation, with previous work showing that alteration of Phe108 can affect ATG12 stability and conjugation to ATG5 in mouse embryonic fibroblasts.[44] While the Tyr120 of ATG12 is relatively close to ATG5, it is not adjacent to its surface, suggesting a less damaging effect on heterodimer formation. Among over 2,000 ATG12 orthologs, Tyr120 is conserved among 44% of sequences, whereas Ala138 is nearly invariant at 89% of sequences (highlighted in Figure 1E).[43] The p.Tyr120Ser and p.Ala138Val substitutions may have an impact on ATG12 interaction with both ATG5 and ATG3. Relevant to this, a substitution at Ala138 (i.e., p.Ala138Arg) showed a minor impairment of ATG12-ATG5-ATG16L1 binding to ATG3 and abolished LC3 lipidation *in vitro*.[44]

ATG12 variants impair autophagy flux and intracellular degradation in subject-derived fibroblasts

Following the results on ATG12 interactions with its binding partners, we performed functional assessment of autophagy by immunoblotting for autophagy-related proteins in control and available *ATG12* subject-derived fibroblasts. Under basal conditions, both S2 and S3 showed stable levels of the core autophagy effectors ATG7 and ATG3, along with stable levels of SQSTM1/p62, an autophagy receptor and substrate (Figure 3A). S2, however, exhibited a clear loss of the ATG12-ATG5 dimer, along with no detectable ATG12 monomer, likely indicating that decrease in the ATG12-ATG5 conjugate is due to a loss of ATG12. Western blot analysis also demonstrates no detectable additional ATG12 species are produced by the c.363+3A>T putative splice variant (Figure S1). In contrast, cells from S3 revealed stable levels of the ATG12-ATG5 conjugate and ATG12 monomer. Further probing identified the presence of unconjugated ATG5 monomer in S2 only (Figure 3A).

Furthermore, we performed a comparative assessment of autophagy flux. Cells were cultured in standard media or under starvation conditions (HBSS) to induce autophagy. We monitored LC3 turnover using a non-saturating concentration of the vacuolar type H^+-translocating ATPase inhibitor $BafA_1$ for 3 h to block autophagic flux at a late stage.[45,46] Under non-starvation conditions, a 2 nM concentration of $BafA_1$ resulted in a minimal increase in the lipid-conjugated form of LC3, LC3-II, indicating the dose was non-saturating for both the control and subject-derived fibroblasts (Figure 3B). Under starvation conditions, a 2 nM $BafA_1$ treatment resulted in a robust increase in LC3-II in the control and S2 fibroblasts; however, the LC3-II increase was markedly reduced in S3 fibroblasts compared to the control and S2 cells. In addition, a saturating dose of 100 nM $BafA_1$ resulted in further accumulation of LC3-II, with S2 fibroblasts being comparable to

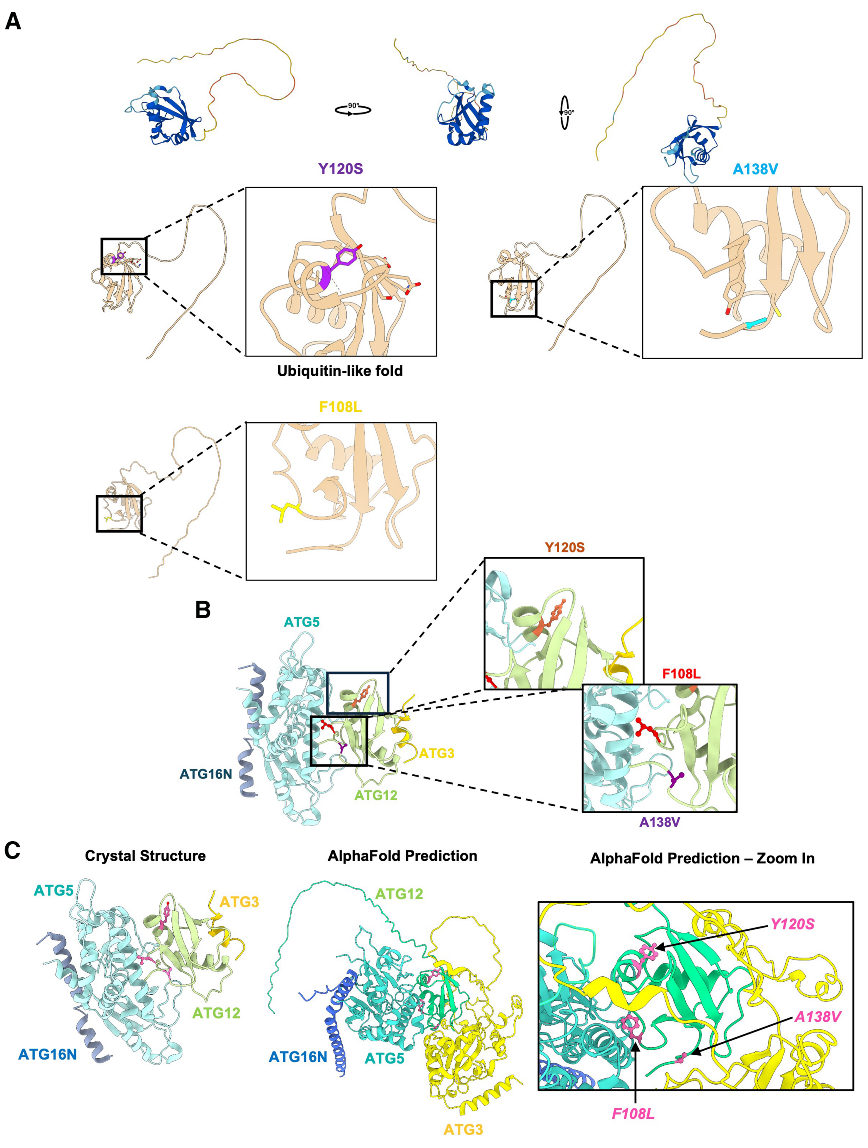

Figure 2. *In silico* structural analysis of ATG12 variants illustrating the effect on complex formation
(A) AlphaFold prediction of ATG12 structure (AlphaFold:O98417), in orthogonal views. Insets: position of p.Tyr120Ser (Y120S) variant in the ubiquitin fold of ATG12 is shown in purple. Position of p.Ala138Val (A138V) variant is shown in blue. Position of the p.Phe108L (F108L) variant is shown in yellow.
(B) Reported crystal structure of the ATG12-ATG5-ATG16N-ATG3 complex (PDB: 4NAW) showing the proximity of the two missense variants in this study to the interface between ATG12 and ATG5.
(C) Comparison of the existing crystal structure of the ATG12-ATG5-ATG16N-ATG3 complex and our expanded AlphaFold model with the two missense variants annotated in pink. The expanded AlphaFold model shows the full-length ATG3 sequence (yellow) wrapped around ATG12 (green), placing the variants in close proximity to the interfaces with both ATG5 and ATG3.

the control. While 100 nM $BafA_1$ increased LC3-II accumulation in S3 fibroblasts, it was still reduced compared to the control and S2. The levels of SQSTM1/p62 did not change in both S2 and S3 fibroblasts and decreased after starvation, suggesting autophagy was suppressed only modestly, even in S3 cells. To complement these findings, we also directly measured non-selective autophagy flux using the classical LDH sequestration assay.[29] Bulk autophagic sequestration was decreased in cells from S3, while flux appeared unaffected in cells from S2 (Figure 3C).

ATG12 p.Phe108Leu and p.Ala138Val variants exhibit modest autophagy defects, while p.Arg79* and c.363+3A>T variants exhibit profound autophagy defects

To investigate the effects of the subject's ATG12 variants on autophagy, we conducted rescue experiments. *ATG12* KO HeLa cells were generated using the CRISPR-Cas9 system, and successful KO was confirmed by DNA sequencing (Figure S2A). Next, the autophagy flux reporter HaloTag–LC3 and each of the 3xHA-ATG12 variants (WT, p.Phe108Leu, p.Tyr120Ser, p.Arg79*, p.Ala138Val, and c.363+3A>T) were stably expressed (Figure 4A).[32] We inserted intron 3 into all the *ATG12* cDNAs to evaluate a potential splicing defect of v.363+3A>T. The mRNA expression levels of the corresponding *ATG12* variants are generally comparable across the cell lines (Figure S2B). Significant amounts of unconjugated ATG12 of the WT and p.Ala138Val were detected, indicating the overexpression of these constructs after successful splicing. The levels of the ATG12-ATG5 conjugate were similar to the endogenous levels, likely because the amount of ATG5 is rate limiting. The ATG12-5 conjugate observed in the sample of ATG12 p.ArgR79* may be the result of stop-codon (TGA) readthrough. The levels of the p.Phe108Leu and p.Tyr120Ser were slightly reduced, while those of p.Arg79* and c.363+3A>T were profoundly reduced (Figures 4B and 4C). These data suggest that the p.Phe108-Leu and p.Tyr120Ser variants destabilize ATG12, although we cannot fully exclude the possibility of decreased translational efficiency. Furthermore, the minimal expression of the intron variant c.363+3A>T confirmed impaired splicing.

Next, we evaluated the autophagic flux of these rescued cells.[47] SQSTM1/p62, a well-established selective receptor and substrate of autophagy, accumulated in *ATG12* KO cells but not in cells rescued with WT ATG12.[48] The expression of SQSTM1/p62 was also returned to basal

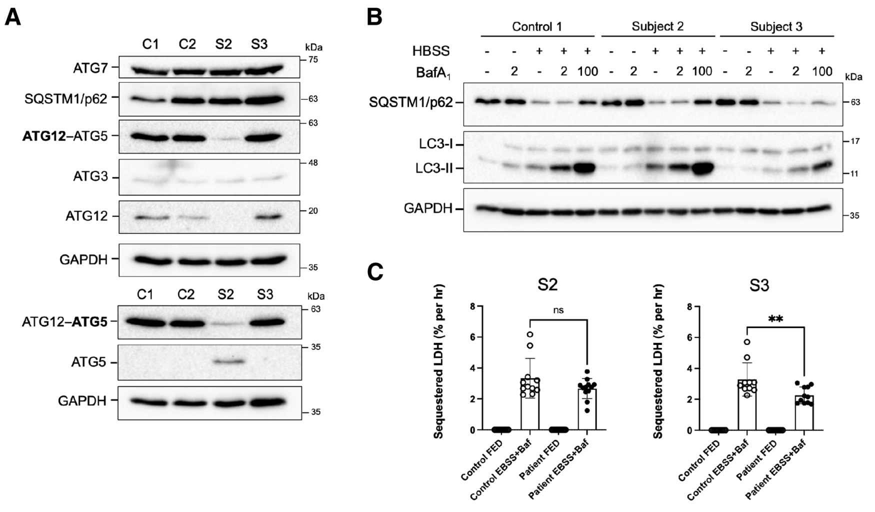

Figure 3. Immunoblot analysis of subject-derived fibroblasts shows decreased ATG12 levels or altered autophagy flux

(A) Immunoblot of steady state levels of autophagy-related proteins in controls (C1 and C2) and subject-derived fibroblasts (S2 and S3). Immunoblots show decreased ATG12-ATG5 conjugate and loss of unconjugated ATG12 monomer in cells from S2 but not S3. Additional blots show the presence of unconjugated ATG5 in S2 but not in S3. Labels in bold denote the antibody used for detection of the conjugate ($n = 3$).

(B) Immunoblot of autophagy flux assay. Subject-derived cells were grown under basal conditions or starvation conditions (HBSS) to induce autophagy and treated with 2 nM or 100 nM $BafA_1$ to block late-stage autophagy for 3 h before harvesting to probe the rate of LC3-II production. S2 fibroblasts showed minimal change, while S3 fibroblasts showed attenuation of LC3-II production compared to the control fibroblasts. ($n = 3$).

(C) Quantification of LDH sequestration per hour under basal or starvation conditions (EBSS) with lysosome acidification inhibited by 100 nM $BafA_1$ for 3 h. Only S3 cells showed a significant decrease in bulk autophagic sequestration ($n = 3$) (**$p < 0.01$ ordinary one-way ANOVA and Sidak's multiple comparison test, error bars indicate SD).

levels in cells expressing p.Phe108Leu, p.Tyr120Ser, and p.Ala138Val but remained elevated in cells expressing p.Arg79* or c.363+3A>T (Figures 4B and D). LC3 lipidation was rescued in cells expressing p.Tyr120Ser, whereas it was partially impaired in cells expressing p.Phe108Leu or p.Ala138Val and was not detected in cells expressing p.Arg79* or c.363+3A>T (Figures 4B and 4E). Since LC3-II abundance reflects the balance between LC3 lipidation and delipidation, and ATG12 is involved only in lipidation, the LC3-I:LC3-II ratio could differ between subject-derived fibroblasts (Figure 3B) and HeLa cells reconstituted with the respective variants (Figure 4B). To quantify autophagic flux more precisely, we performed the HaloTag-LC3 processing assay.[32] When HaloTag-LC3 is delivered to lysosomes via autophagy, it is efficiently degraded. However, after binding to its ligands, such as the tetramethylrhodamine (TMR)-conjugated ligand, HaloTag becomes resistant to lysosomal degradation, allowing the HaloTag fragment to accumulate depending on autophagic activity (Figure 4F). The HaloTag fragment was not detected in *ATG12* KO cells but was detected in cells expressing WT ATG12 after 6-h starvation (Figures 4G and 4H). The amount of the HaloTag fragment decreased in cells expressing p.Phe108-Leu or p.Ala138Val and was virtually undetectable in cells expressing p.Arg79* or c.363+3A>T. The c.235C>T (p.Arg79*) nonsense variant occurs within exon 2 of 4 and crucially >50 nucleotides upstream of the final exon-exon junction; according to the established model of nonsense-mediated decay, this variant is therefore predicted to initiate nonsense-mediated mRNA decay (NMD) and degradation of the variant-associated transcript. These results suggest that p.Phe108Leu and p.Ala138Val exhibit significantly reduced autophagic activity, and p.Arg79* and c.363+3A>T exhibit virtually no autophagic activity.

In contrast, cells expressing p.Tyr120Ser demonstrated normal HaloTag processing (Figures 4G and 4H), suggesting intact autophagic activity in this experimental setting. However, given that p.Tyr120Ser levels decreased in subject-derived fibroblasts (obtained from S2), p.Tyr120Ser may generate an autophagy defect at the endogenous expression level, which could be overcome by its overexpression.

Disease-associated Atg12 variants impair Atg8 lipidation and autophagy flux under acute nitrogen starvation in yeast

Our analyses of autophagic flux in subject-derived primary cells revealed subtle biochemical deficits. As *ATG12* and the autophagy process are evolutionarily conserved, we were able to use complementation studies in *S. cerevisiae* expressing homologous *Atg12* variants to assess their effects on autophagy *in vivo*. Sequence alignments demonstrated $ATG12^{Phe108}$, $ATG12^{Tyr120}$, and $ATG12^{Ala138}$ of the human ATG12 correspond to $ATG12^{Phe154}$, $ATG12^{Trp166}$, and $ATG12^{Ala184}$, respectively, of yeast Atg12 (Figure 5A). We constructed *atg12Δ* yeast cells and complemented them with 3xHA-Atg12, 3xHA-$Atg12^{F154L}$, 3xHA-$Atg12^{W166S}$, or 3xHA-$Atg12^{A184V}$ via integrative plasmids. As Atg12 can conjugate with Atg5 and function as an E3-like enzyme to catalyze the lipidation reaction of Atg8, we first examined whether starvation-induced Atg8 lipidation was affected by these mutations. The attachment of the lipid molecule PE at the C-terminal end of Atg8 results in faster electrophoretic mobility in urea-SDS-PAGE gels relative to the nonlipidated form. No Atg8 lipidation occurred in the empty vector (−) transformant, whereas robust Atg8 lipidation was observed in the *atg12Δ* cells transformed with the 3xHA-Atg12 (WT) plasmid (Figure 5B). The 3xHA-$Atg12^{A184V}$ mutant

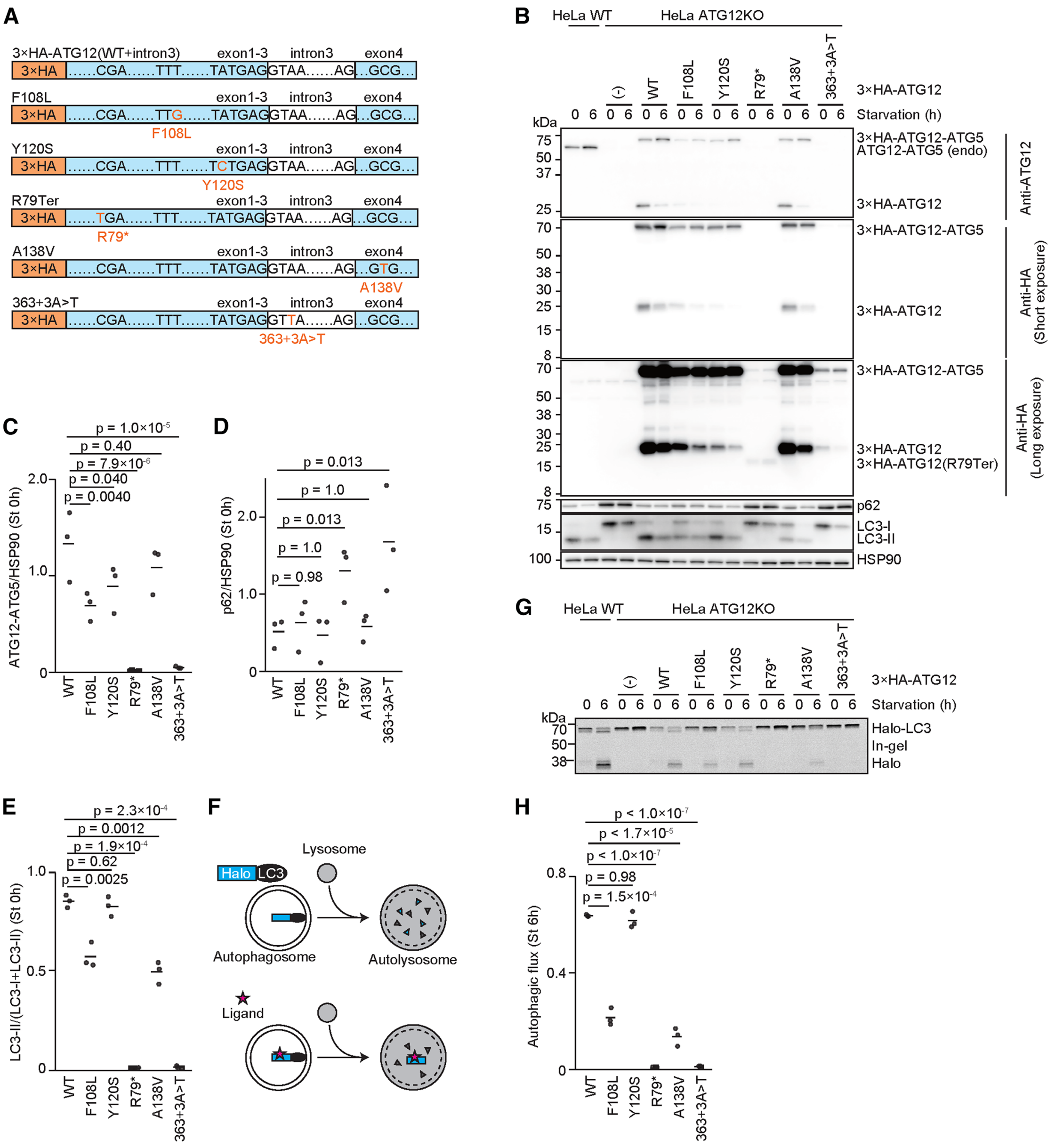

Figure 4. Autophagic activity of the ATG12 variants
(A) A structural diagram of the plasmids encoding 3xHA-tagged wild-type (WT) human ATG12 and its variants containing intron 3.
(B) Levels of endogenous ATG12, exogenous 3xA-ATG12 variants, LC3, and p62 before and after 6-h starvation.
(C) Quantification of the levels of ATG12-ATG5 in (B). Horizontal lines indicate the means, and dots indicate the individual data from three independent experiments. Data were statistically analyzed using one-way ANOVA with Tukey test.
(D) Quantification of the levels of p62 in (B). Horizontal lines indicate the means, and dots indicate the individual data from three independent experiments. Data were statistically analyzed using one-way ANOVA with Tukey test.
(E) Quantification of the levels of LC3 in (B). Horizontal lines indicate the means, and dots indicate the individual data from three independent experiments. Data were statistically analyzed using one-way ANOVA with Tukey test.
(F) Schematic representation of the HaloTag-LC3 processing assay. HaloTag, which is normally degraded in lysosomes, becomes stable after ligand binding.
(G) In-gel fluorescence detection of the HaloTag fragment in HaloTag-LC3-expressing HeLa cells pulse-labeled with 100 nM TMR conjugated ligand for 20 min. Cells were starved for 6 h.
(H) Quantification of the results shown in (G). $HaloTag^{TMR}$ band intensity was normalized by the sum of the band intensities ($HaloTag^{TMR}$-LC3 and $HaloTag^{TMR}$). Horizontal lines indicate the means, and dots indicate the individual data from three independent experiments. Data were statistically analyzed using one-way ANOVA with Tukey test.

partially rescued Atg8 lipidation defects, but to a lesser extent than 3xHA-Atg12. The 3xHA-$Atg12^{F154L}$ and the 3xHA-$Atg12^{W166S}$ mutants caused more severe defects in Atg8 lipidation.

To quantitatively characterize the effects of these mutations on bulk autophagy flux, we performed the GFP-Atg8 processing assay: in brief, a portion of GFP-tagged Atg8 bound to the inner side of the autophagosome is delivered

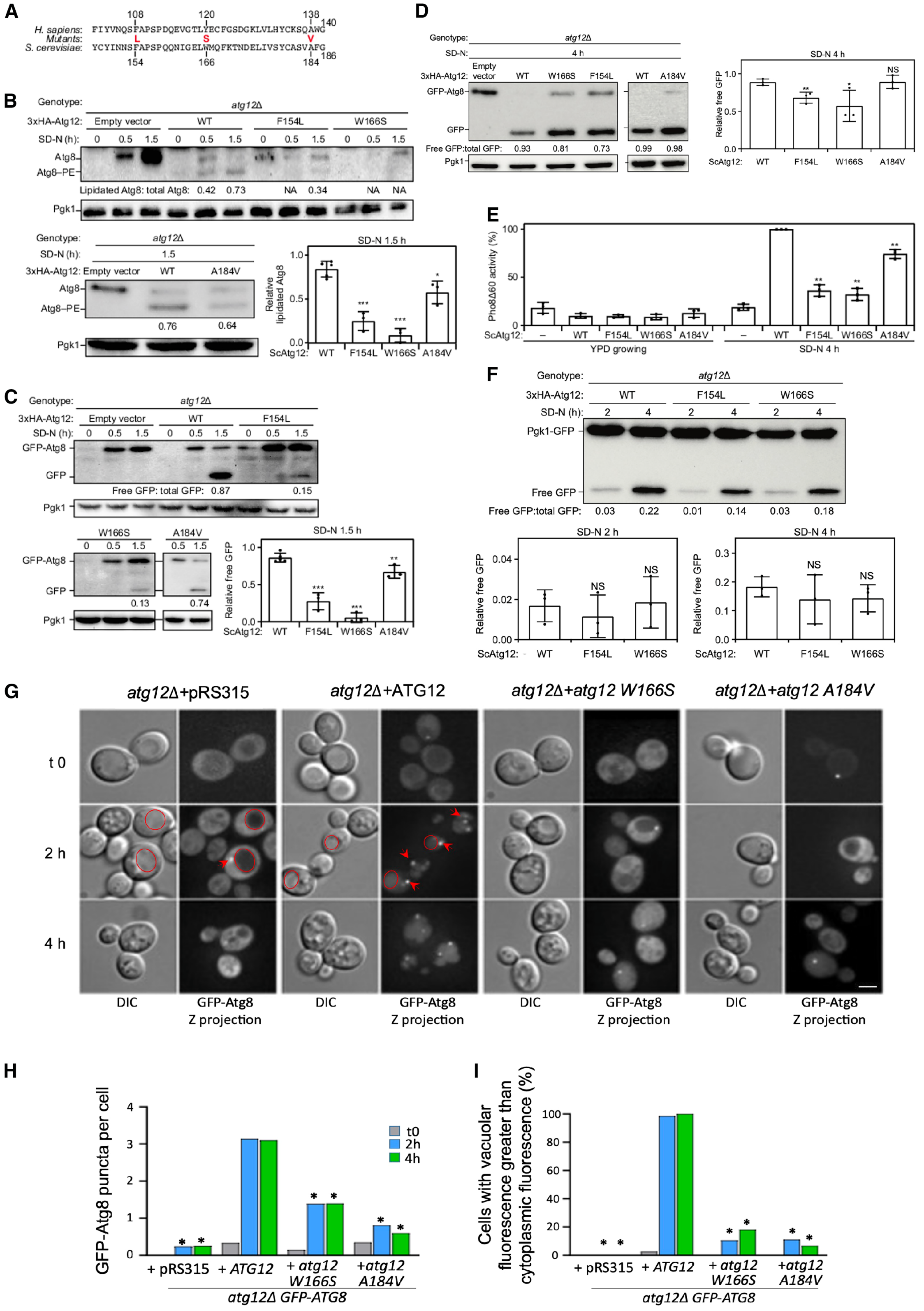

Figure 5. Disease-associated mutations of *Atg12* impair Atg8 lipidation and autophagy flux under acute nitrogen starvation in yeast

(A) Alignment of the human (UniProtKB: O98417) and yeast ATG12 (UniProtKB: P38316) sequences covering the amino acids F154L, W166S, and A184V.

(B) WLY176 *atg12*Δ cells transformed with *pRS406* empty vector, *pRS406-ATG12p-3xHA-ATG12*, *pRS406-ATG12p-3xHA-ATG12[F154L]*, *pRS406-ATG12p-3xHA-ATG12[W166S]*, or *pRS406-ATG12p-3xHA-ATG12[A184V]* were grown to mid-log phase in YPD (0 h), centrifuged, and resuspended in SD-N medium for the indicated times. Cell lysates were prepared, subjected to 18%

(legend continued on next page)

to the vacuole via autophagy. The GFP is relatively resistant to vacuolar hydrolytic enzymes in comparison to Atg8, and the free GFP can be resolved and detected by western blot. The ratio between free GFP versus total GFP reflects the flux of vacuolar delivery of the autophagosomes. Under acute nitrogen starvation (SD-N 0.5 h and SD-N 1.5 h), there was no GFP-Atg8 processing signal in the empty vector transformants, but the free GFP cleavage from the GFP-Atg8 fusion protein was abundant in the *atg12*Δ cells transformed with the WT 3xHA-Atg12 plasmid (Figure 5C). In contrast, the 3xHA-Atg12^{F154L}, or 3xHA-Atg12^{W166S} transformants only partially rescued the GFP-Atg8 processing defect in the *atg12*Δ cells.

We observed that, during prolonged nitrogen starvation (SD-N, 4 h), the negative effects of the mutations became milder (Figure 5D). We reasoned that these mutations only partially affect the functions of the conjugating enzyme. As the starvation time increases, the total autophagy flux becomes similar to that of the WT, which has reached a plateau.

In the second autophagy flux assay, Pho8Δ60 is an engineered form of the Pho8 phosphatase whose vacuolar delivery is autophagy dependent. Once entering vacuoles, the Pho8Δ60 propeptide is cleaved and activated. Pho8Δ60 activity was low under nutrient-rich conditions among all three transformants (Figure 5E). After 4 h in the nitrogen-starvation medium, robust Pho8Δ60 activity was induced in the 3xHA-Atg12 transformants but not the empty vector transformants. The Pho8Δ60 activity was lower in all three mutants, supporting the hypothesis that these mutations in Atg12 impaired autophagy flux.

We also performed a Pgk1-GFP processing assay to further examine the effects of the mutations on autophagic cargo degradation. However, these mutations did not affect Pgk1-GFP processing (Figure 5F), likely because its degradation primarily occurs during prolonged starvation, when the autophagy flux defect caused by the mutations is less pronounced.

Autophagic activity can also be quantitatively measured using microscopy. GFP-Atg8 delivery to the vacuole via autophagosomes results in GFP puncta accumulating inside the vacuole, indicating autophagic activity. Punctate GFP signals indicative of autophagosomal structures were still able to form in strains harboring Atg12^{W166S} and Atg12^{A184V} variants; however, their number was greatly decreased (Figures 5G and 5H). Additional quantification of GFP presence in the vacuole also confirmed a reduction in the delivery of GFP to the degradative compartment (Figure 5I).

Loss of *atg12* function in zebrafish causes developmental delay, impaired brain function, and pre-adult lethality

To investigate the *in vivo* function of ATG12, we generated a KO in the zebrafish ortholog, *atg12*, using CRISPR-Cas9-mediated mutagenesis. A 10-bp deletion within exon 2 of the *atg12* gene was identified and confirmed by Sanger sequencing (Figure 6A). This frameshift mutation produces a premature termination codon that is predicted to trigger NMD, resulting in a loss-of-function allele. Morphological analyses revealed that *atg12*$^{-/-}$ larvae were morphologically indistinguishable from their *atg12*$^{+/+}$ siblings at 5 dpf. However, as development progressed, *atg12*$^{-/-}$ larvae exhibited developmental delay, and, by 15 dpf, they were notably smaller than their WT siblings (Figure 6B). Genotyping analyses showed that *atg12*$^{-/-}$ larvae were present at Mendelian ratios at 5 dpf but progressively declined by 60 dpf, indicating that homozygous mutants fail to survive to adulthood (Figure 6C). These findings suggest that *atg12* is essential for post-larval growth and viability in zebrafish.

To assess the impact of *atg12* deficiency on neurodevelopment in zebrafish, we evaluated behavior assays to measure locomotor responses in the light-dark cycle (LDC) and visual startle response (VSR) assays. In the LDC assay, WT larvae displayed robust locomotor activity characterized by increased distance traveled during dark phases and subsequent habituation across cycles (Figure 6D). In contrast, *atg12*$^{-/-}$ mutants exhibited markedly reduced locomotor activity throughout both light and dark phases. Consistent with these findings, *atg12*$^{-/-}$

urea-SDS-PAGE, and analyzed by western blot. The ratio of lipidated Atg8 to total Atg8 (lipidated Atg8 plus nonlipidated Atg8) was quantified to indicate the Atg8 lipidation level.
(C and D) Cell lysates were prepared, subjected to 10% SDS-PAGE, and analyzed by western blot. The ratio of free GFP to total GFP (free GFP plus GFP-Atg8) was quantified to indicate autophagy flux. Pgk1 was used as a loading control.
(E) Cell lysates were prepared and analyzed by the Pho8Δ60 assay. Pho8Δ60 activity was normalized to WLY176 *atg12*Δ cells transformed with *pRS406-ATG12p-3xHA-ATG12* in SD-N for 4 h (set to 100%).
(F) SEY6210 *atg12*Δ cells transformed with *pRS406* empty vector, *pRS406-ATG12p-3xHA-ATG12*, *pRS406-ATG12p-3xHA-ATG12[F154L]*, *pRS406-ATG12p-3xHA-ATG12[W166S]*, or *pRS406-ATG12p-3xHA-ATG12[A184V]* were grown to mid-log phase in YPD (0 h), centrifuged, and resuspended in SD-N medium for the indicated times. Cell lysates were prepared, subjected to 10% SDS-PAGE, and analyzed by western blot. The ratio of free GFP to total GFP (free GFP plus Pgk1-GFP) was quantified to indicate autophagic cargo degradation. Summary data are presented as the mean ± SD. *$p < 0.05$, **$p < 0.01$, ***$p < 0.001$; NS, not significant; Student's t test. For all western blot and Pho8Δ60 experiments, $n = 3$–5.
(G) GFP-Atg8 live imaging in an *atg12*Δ strain transformed with plasmids expressing WT or mutant *ATG12*. Cells were collected before (t0) and at 2 and 4 h after nitrogen starvation. Red arrows indicate autophagosomes or phagophores and red circles indicate the yeast vacuole (scale bar, 3 μm).
(H) Average GFP-Atg8 puncta per cell. Significance relative to the *atg12*Δ strain expressing the *ATG12* plasmid (*$p < 0.001$ Kruskall-Wallis test) ($n = 3$).
(I) Percentage of cells with vacuolar fluorescence greater than the cytoplasmic fluorescence. Significance relative to the *atg12*Δ strain expressing the *ATG12* plasmid (*$p = 0.001$ Kruskall-Wallis test) ($n = 3$).

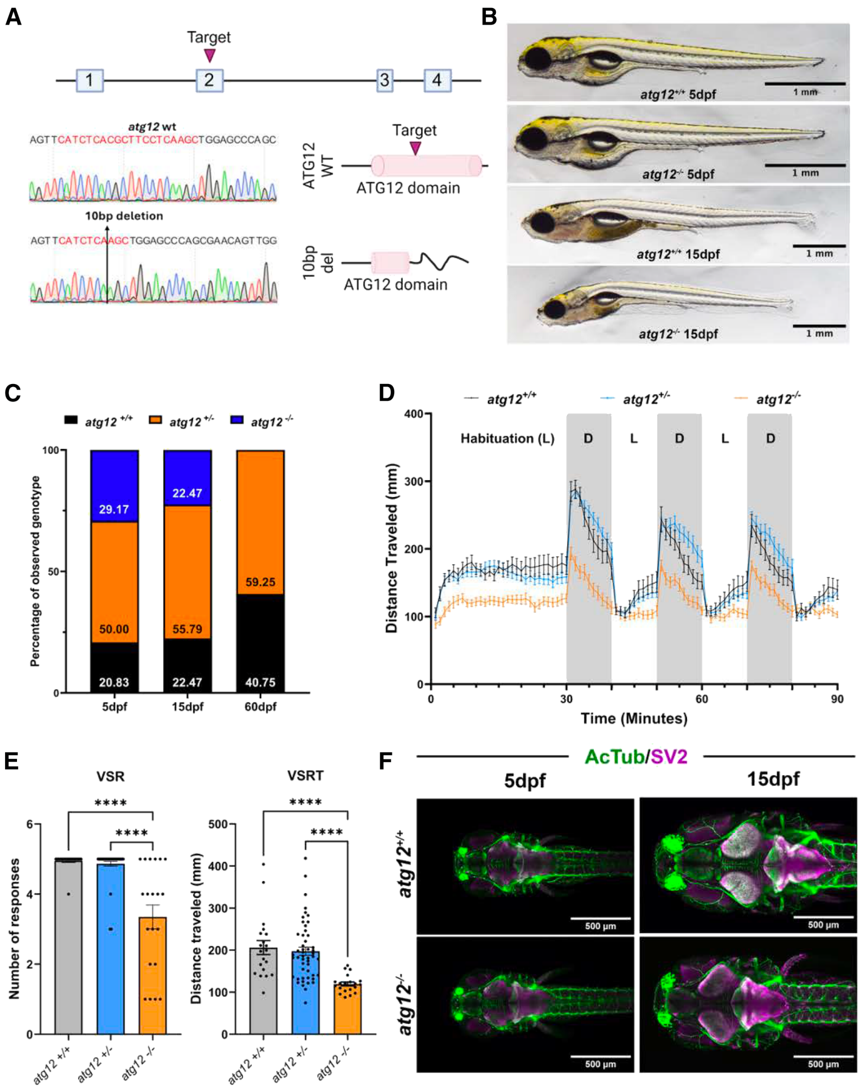

Figure 6. Loss of *atg12* impairs zebrafish development, brain function, and survival

(A) CRISPR-Cas9-mediated mutagenesis generated a 10-bp deletion within exon 2 of *atg12*, confirmed by Sanger sequencing. The resulting frameshift is predicted to truncate the ATG12 domain, producing a loss-of-function allele.

(B) Bright-field images of *atg12*+/+ and *atg12*−/− larvae at 5 and 15 dpf. Mutants are morphologically indistinguishable from wild type at 5 dpf but show developmental delay and reduced body size by 15 dpf. Scale bars, 1 mm.

(C) Genotypic distribution of *atg12*+/+, *atg12*+/−, and *atg12*−/− larvae at 5, 15, and 60 dpf. Homozygous mutants were present at Mendelian ratios at 5 dpf but progressively declined by 60 dpf, indicating decreased post-larval survival.

(D) Locomotor activity during alternating light (L) and dark (D) phases. WT larvae exhibited robust increases in movement following light-to-dark transitions and subsequent habituation, whereas *atg12*−/− mutants showed significantly reduced activity throughout both phases.

(E) Quantification of visual startle responses (VSRs). Both the number of responses (VSRs) and total distance traveled (VSRT) were significantly reduced in $atg12^{-/-}$ larvae compared with WT and heterozygous siblings. Data represent mean ± SEM. ****$p < 0.0001$ by one-way ANOVA with *post hoc* comparisons.

(F) Light-sheet images of acetylated tubulin (AcTub, green) and synaptic vesicle protein 2 (SV2, magenta) in $atg12^{+/+}$ and $atg12^{-/-}$ larvae at 5 and 15 dpf. Mutants exhibit reduced brain size reduced synaptic intensity at 15 dpf. Scale bars, 500 µm.

larvae demonstrated significantly attenuated visual startle responses, with both the number of responses (VSR) and total distance traveled (VSRT) significantly reduced compared to WT controls (Figure 6E). Together, these results indicate that loss of Atg12 impairs sensorimotor function and arousal responses, leading to reduced locomotor output and impaired responses to changes in visual stimuli.

To further examine the structural basis of these functional deficits, we analyzed brain development using immunohistochemistry. Larvae at 5 and 15 dpf were stained for acetylated tubulin (AcTub) and the synaptic vesicle marker SV2. At 5 dpf, $atg12^{-/-}$ larvae showed no obvious differences in brain morphology or axonal organization compared to WT siblings. However, by 15 dpf, mutants exhibited disorganized axonal tracts, reduced synaptic labeling, and smaller overall brain size (Figure 6F), suggesting defects in neuronal connectivity and synapse formation.

In summary, our data demonstrate that *Atg12* is required for normal growth, brain development, and neural function in zebrafish. These findings provide functional evidence linking *Atg12* disruption to impaired neurodevelopment, supporting a potential role for *Atg12* dysfunction in the pathophysiology of human neurodevelopmental disorders.

Discussion

Here, we report six individuals from five unrelated families harboring variants in the core autophagy gene *ATG12*, a gene not previously associated with a human phenotype. The individuals share clinical features of ataxia and global developmental delay with the neuroradiological finding of profound cerebellar hypoplasia. For comparison with the phenotypes of previously reported individuals with *ATG5* and *ATG7* pathogenic variants, see Table S5.

Our *in silico* analyses using published X-ray crystal structures, combined with our AlphaFold structural modeling, suggest that the p.Tyr120Ser and p.Ala138Val variants

map to a ubiquitin-like fold in ATG12, with the p.Phe108-Leu variant being previously shown to affect ATG12 conjugation and function.[44] While we observed loss of the stable ATG12-ATG5 complex in S2 cells, this was not observed in S3 cells, although only S3-derived fibroblasts showed reduced autophagic flux. Additionally, *ATG12* KO HeLa cells and *atg12*-null yeast cells are unable to fully recover autophagy when complemented with equivalent missense ATG12 (except p.Tyr120Ser) and *Atg12* variants, respectively, suggesting the identified variants have a negative effect *in vivo*. Regardless, while the human *ATG12* and yeast *Atg12* variants retain partial function, the mild autophagy attenuation observed across the multiple functional assays strongly suggests that these variants are detrimental to autophagy. This finding, combined with the neurological phenotypes observed in both families, suggests that the autophagy impairment stemming from *ATG12* variants is incompatible with sustaining neural integrity in humans. Zebrafish functional studies identify a critical role for Atg12 in zebrafish development and neural function. Disruption of *atg12* through CRISPR-Cas9-mediated mutagenesis resulted in a loss-of-function allele that caused profound post-larval growth delay, reduced locomotor activity, and pre-adulthood lethality. Despite normal early morphology at 5 dpf, $atg12^{-/-}$ larvae exhibited progressive defects in neuronal organization, reduced synaptic labeling, and impaired sensorimotor responses. In humans, deficits in these processes are characteristic of neurodevelopmental disorders, including ataxia, intellectual disability, and epilepsy, where alterations in sensory responsiveness, motor coordination, and habituation are commonly observed. Thus, the zebrafish *atg12* mutants provide a tractable *in vivo* model to investigate how defective autophagy contributes to these phenotypes.

Decades of work across various model systems have elaborated the intricate molecular regulation and function of canonical macroautophagy. As part of this work, numerous KO mouse models of various *ATG* genes have been produced and characterized, including models of *Atg12*, *Atg5*, *Atg3*, and *Atg7*.[13–15,49] Global KO models of these genes involved in the ATG conjugation system result in perinatal lethality, implying that deleterious variants in these genes are incompatible with life. However, in addition to these results involving *ATG12*, recent publications from our group and others have described cases of individuals harboring pathogenic variants in *ATG5* and *ATG7* surviving past birth, with some affected individuals reaching ages toward the population's life expectancy.[8–10] This observation suggests a potential for supplemental or compensatory pathways in humans that may not be present in shorter-lived model organisms such as mice. It is important to remember that previous work has suggested autophagy is generally essential in mammals; mutant alleles resulting in severe defects may therefore result in non-viable embryos, favoring the detection of mutations that result in more subtle phenotypes.[50] Nevertheless, the neuropathology observed in a pre-clinical CNS-specific *atg7* conditional KO model, together with those reported here, underscores the essential role of autophagy in maintaining neural integrity in mammals.[9–11]

An additional feature of interest in these *ATG12* cases is the consistency in neuroradiological findings with those reported in individuals with *ATG7* and *ATG5* mutations (Table S5).[8,9] The hypoplasia of the corpus callosum observed in S1, S3, S4, and S5 is also consistent with the *ATG7* and *ATG5* cohorts, as is the cerebellar hypoplasia observed.[8,9,51] It is unclear why these areas appear to be most susceptible to damage, but the consistency across the cohort would suggest these areas are more reliant on autophagy during development. More specifically these regions, especially the cerebellum, may be more reliant on mitophagy, a form of selective autophagy specific to mitochondria, during development. Previous work using the *mito*-QC reporter mouse has demonstrated that cerebellar Purkinje cells exhibit high levels of mitophagy in the cerebellum of mice.[52] Additionally it has been demonstrated that mitophagy rates in cerebellar Purkinje cells and external granule cells of aging mice increase; however, *auto*-QC analysis showed that bulk autophagy does not follow the same trend.[53] This finding demonstrates the disparity between autophagy and mitophagy in cerebellar cell types and may hint that certain forms of selective autophagy are the predominant drivers for the specificity of the phenotypes associated with congenital *ATG* disorders. Similar work investigating the selective autophagy rates in contrast to bulk autophagy in the developing brain may help to elucidate this.

Given the similarities between the various *ATG* cohorts (Table S5), it would be interesting to ascertain whether a generalized autophagy modulation therapy would be effective for all three of the described disorders or whether they would require more specific interventions, such as gene therapy approaches. However, given the clear developmental component in these infantile-onset disorders, further insights are needed to identify intervention points that could offer maximum benefit.

The range of phenotype severity in the cohort is also interesting. Fibroblasts from S2 were found to have LC3 lipidation comparable to that of the control in autophagy flux assays, despite their loss of the ATG12-ATG5 complex, likely due to the minimal amount of the ATG12-ATG5 conjugate required for efficient lipidation of ATG8 proteins.[54] However, the corresponding subjects were unable to survive past early childhood, with S1 dying of intractable infantile spasms and epileptic encephalopathy at 12 months, and sibling S2 also being diagnosed with epileptic encephalopathy and dying in early childhood. In contrast, S3 was found to maintain their ATG12-ATG5 conjugate, but S3-derived fibroblasts had a severe reduction in LC3-I lipidation when analyzed by an autophagy flux assay. Despite this, S3 appears to have a less severe neurological phenotype but with the complication of kidney disease and other

non-neurological phenotypes. The kidney-disease phenotype was also observed in his sibling, not available for genetic testing, suggesting he too may have been affected with the same genetic variants. The seemingly paradoxical relationship between biochemical defect and phenotype is not without precedent. Of the previously described cohort with *ATG7* variants, S1 and S2 (segregating loss-of-function variants) had the most severe biochemical defect with undetectable levels of ATG7 and no detectable LC3-II production, yet presented with the mildest phenotype.[8] In contrast, S3 and S4 (segregating missense variants) had detectable levels of ATG7 and LC3-II production by immunoblot yet presented with a more severe neurological phenotype.[8]

This phenotypic disparity does highlight a limitation of our zebrafish models, this being that the *atg12* KO allele directly recapitulates the loss-of-expression variants but does not model the partial loss of function caused by missense variants. The functional consequences of missense variants are addressed by our yeast complementation and HeLa cell rescue experiments, which demonstrate variant-specific gradients of autophagy impairment. However, generation of knockin zebrafish models expressing missense variants identified in affected individuals would further elucidate genotype-phenotype relationships and variant-specific disease mechanisms.

In family 2, both S3 and an untested sibling presented with kidney disease. This phenotype could represent a less common manifestation of ATG12 dysfunction or may have an unrelated genetic etiology, especially considering parental consanguinity in this family, which increases the risk for recessive disorders. Despite extensive analysis, WES did not reveal any putative pathogenic variants that could explain the kidney phenotype.

It is possible that the early lethality observed in family 1 may have prevented a kidney-disease phenotype from developing; however, its absence from other members of this family and the *ATG7* and *ATG5* cohorts would appear to make this less likely. It is hoped that this report of *ATG12*-related pathology will result in *ATG12* featuring in prospective diagnostic testing algorithms, and further expansion of the genotype-phenotype correlation may help elucidate the potential renal involvement as additional families are diagnosed and reported. Similarly, for early lethality, although S1 and S2 had a rapidly progressive and fatal disease course, only one other individual with a pathogenic *ATG* variant is reported to have the same severe phenotype, from the *ATG7* cohort, while other individuals with *ATG* variants have a milder disease course.[8] It is likely that genetic diagnoses of *ATG* disorders will increase with the widespread use of next-generation sequencing in the diagnostic process.

Another phenotypic similarity to the *ATG7* cohort is optic atrophy, found in 7/9 investigated individuals, as in S3.[8] Nystagmus in S2 and the deceased sister of S3 may have resulted from optic atrophy, although this remains uncertain. Sensorineural deafness is also reported in 2/12 individuals in the *ATG7* cohort, as in S3 described here (Table S5).[8] Together, this demonstrates that, while the main overlapping phenotypes remain the cerebellar hypoplasia and ataxia, there are other similarities between *ATG*-related disorders, which will no doubt expand with the identification of new affected individuals.

The phenotypes of individuals with pathogenic variants in the *ATG5*, *ATG7*, and *ATG12* genes are clearly distinct from the phenotypes of beta-propeller protein-associated neurodegeneration (BPAN) individuals with mutations in *WDR45* (*WIPI4*; MIM: 300526).[55] *WDR45* (*WIPI4*) is one of the four human homologs of yeast Atg18, and it functions with ATG2. BPAN is characterized by iron deposition in the globus pallidus and the substantia nigra and presents with non-progressive psychomotor retardation during childhood and rapidly progressing Parkinson-like symptoms in adulthood. Since these symptoms differ significantly from those of diseases caused by pathogenic variants in genes related to the ATG conjugation system, it is possible that the impairment of non-autophagy functions of WDR45/WIPI4 contributes to the onset of BPAN.[56]

Next-generation-sequencing efforts have been instrumental in revealing novel congenital autophagy-associated defects that were once thought to be incompatible with life.[57] Although still exceedingly rare, our findings extend the repertoire of genetic variants linked to autophagy dysfunction and further emphasize the clinical significance of autophagy for human nervous system health and disease. We anticipate that the discovery and profiling of additional *ATG* variants will pave the way for therapeutic interventions aimed at ameliorating disorders associated with autophagic dysfunction.

Data and code availability

The exome datasets supporting this study have not been deposited in a public repository because of ethical restrictions but are available upon reasonable request. The *ATG12* variants identified have been submitted to ClinVar (https://www.ncbi.nlm.nih.gov/clinvar/) with the accession numbers SCV007432508, SCV007432509, SCV007432510, SCV007432511, and SCV007432512.

Acknowledgments

J.L. is supported by a PhD studentship funded by the Pathological Society. C.L.A. is supported by a National Institute for Health Research Post-Doctoral Fellowship (PDF-2018-11-ST2-021). R.W.T. is funded by the Medical Research Council (MR/W019027/1), the UK NIHR Biomedical Research Centre for Ageing and Age-related disease award to the Newcastle upon Tyne Foundation Hospitals NHS Trust, LifeArc and the UK NHS Highly Specialised Service for Rare Mitochondrial Disorders of Adults and Children. R.W.T. and M.O. receive support from Mito Foundation, the Lily Foundation, and the Pathological Society. M.O. is supported by Fight for Sight and the Academy of Medical Sciences. D.M. is supported by Anders Jahres Fond til Vitenskapens Fremme. E.F. is supported by the Nasjonal Kompetansetjeneste for Sjeldne Diagnoser. N.M. is supported by a Grant-in-Aid for Specially

Promoted Research (22H04919) from the Japan Society for the Promotion of Science. T.G.M. is funded by the Research Council of Finland, Sigrid Jusélius Foundation, University of Helsinki, Novo Nordisk Fonden, the Päivikki and Sakari Sohlberg Foundation, the Jane and Aatos Erkko Foundation and is a Scholar of the FENS-Kavli Network of Excellence. R.L. is funded by INSERM and the Agence National de la Recherche (ANR-23-CE13-0013-01). D.J.K. is supported by NIH grant GM131919. G.K.V. is supported by the Office of Research Infrastructure Programs and NIH (R24OD034438). The views expressed in this publication are those of the authors and not necessarily those of the NHS, the NIHR, or the Department of Health and Social Care.

Author contributions

J.L., S.A., Y.H., F.S., T.E., and M.P. carried out the experimental assessments of autophagy in subject-derived cells and model systems. J.L. and T.J.M. performed structural modeling studies. A.I., E.H., D.M., G.R., P.V., R. Maroofian, H.H., and C.L.A. performed the molecular genetic analyses. D.W. and K.F. undertook and assessed the neuroradiological assessments. H.M., A.B., M.F.S., C.K., E.F., S.N., A.S., R.S., F.R., S.M., V.U., and R. McFarland supported the clinical investigations of all families. J.L., J.J.C., R. Maroofian, W.W.Y., D.J.K., R.L., N.M., T.G.M., M.O., C.L.A., and R.W.T. interpreted all the data. R.W.T., W.W.Y., D.J.K., R.L., N.M., T.G.M., M.O., and C.L.A. supervised the study and acquired funding. J.L., S.A., Y.H., D.J.K., N.M., T.G.M., M.O., C.L.A., and R.W.T. wrote the manuscript. All authors contributed to the revision of the manuscript.

Declaration of interests

The authors declare no competing interests.

Supplemental information

Supplemental information can be found online at https://doi.org/10.1016/j.ajhg.2026.03.002.

Received: October 13, 2025
Accepted: March 2, 2026
Published: March 26, 2026

References

1. Galluzzi, L., Baehrecke, E.H., Ballabio, A., Boya, P., Bravo-San Pedro, J.M., Cecconi, F., Choi, A.M., Chu, C.T., Codogno, P., Colombo, M.I., et al. (2017). Molecular definitions of autophagy and related processes. EMBO J. *36*, 1811–1836. https://doi.org/10.15252/embj.201796697.
2. Antonioli, M., Di Rienzo, M., Piacentini, M., and Fimia, G.M. (2017). Emerging Mechanisms in Initiating and Terminating Autophagy. Trends Biochem. Sci. *42*, 28–41. https://doi.org/10.1016/j.tibs.2016.09.008.
3. Mizushima, N., Noda, T., Yoshimori, T., Tanaka, Y., Ishii, T., George, M.D., Klionsky, D.J., Ohsumi, M., and Ohsumi, Y. (1998). A protein conjugation system essential for autophagy. Nature *395*, 395–398. https://doi.org/10.1038/26506.
4. Ichimura, Y., Kirisako, T., Takao, T., Satomi, Y., Shimonishi, Y., Ishihara, N., Mizushima, N., Tanida, I., Kominami, E., Ohsumi, M., et al. (2000). A ubiquitin-like system mediates protein lipidation. Nature *408*, 488–492. https://doi.org/10.1038/35044114.
5. Mizushima, N. (2020). The ATG conjugation systems in autophagy. Curr. Opin. Cell Biol. *63*, 1–10. https://doi.org/10.1016/j.ceb.2019.12.001.
6. Yang, Y., and Klionsky, D.J. (2020). Autophagy and disease: unanswered questions. Cell Death Differ. *27*, 858–871. https://doi.org/10.1038/s41418-019-0480-9.
7. Lin, T.A., Wu, V.C.C., and Wang, C.Y. (2019). Autophagy in Chronic Kidney Diseases. Cells *8*, 61. https://doi.org/10.3390/CELLS8010061.
8. Collier, J.J., Guissart, C., Oláhová, M., Sasorith, S., Piron-Prunier, F., Suomi, F., Zhang, D., Martinez-Lopez, N., Leboucq, N., Bahr, A., et al. (2021). Developmental Consequences of Defective ATG7-Mediated Autophagy in Humans. EnglandN. Engl. J. Med. Overseas. Ed. *384*, 2406–2417. https://doi.org/10.1056/NEJMoa1915722.
9. Kim, M., Sandford, E., Gatica, D., Qiu, Y., Liu, X., Zheng, Y., Schulman, B.A., Xu, J., Semple, I., Ro, S.-H., et al. (2016). Mutation in ATG5 reduces autophagy and leads to ataxia with developmental delay. eLife *5*, e12245. https://doi.org/10.7554/eLife.12245.
10. Yapici, Z., and Eraksoy, M. (2005). Non-progressive congenital ataxia with cerebellar hypoplasia in three families. Acta Paediatr. *94*, 248–253. https://doi.org/10.1111/j.1651-2227.2005.tb01902.x.
11. Komatsu, M., Waguri, S., Chiba, T., Murata, S., Iwata, J.i., Tanida, I., Ueno, T., Koike, M., Uchiyama, Y., Kominami, E., and Tanaka, K. (2006). Loss of autophagy in the central nervous system causes neurodegeneration in mice. Nature *441*, 880–884. https://doi.org/10.1038/nature04723.
12. Hara, T., Nakamura, K., Matsui, M., Yamamoto, A., Nakahara, Y., Suzuki-Migishima, R., Yokoyama, M., Mishima, K., Saito, I., Okano, H., and Mizushima, N. (2006). Suppression of basal autophagy in neural cells causes neurodegenerative disease in mice. Nature *441*, 885–889. https://doi.org/10.1038/nature04724.
13. Komatsu, M., Waguri, S., Ueno, T., Iwata, J., Murata, S., Tanida, I., Ezaki, J., Mizushima, N., Ohsumi, Y., Uchiyama, Y., et al. (2005). Impairment of starvation-induced and constitutive autophagy in Atg7-deficient mice. J. Cell Biol. *169*, 425–434. https://doi.org/10.1083/jcb.200412022.
14. Kuma, A., Hatano, M., Matsui, M., Yamamoto, A., Nakaya, H., Yoshimori, T., Ohsumi, Y., Tokuhisa, T., and Mizushima, N. (2004). The role of autophagy during the early neonatal starvation period. Nature *432*, 1032–1036. https://doi.org/10.1038/nature03029.
15. Malhotra, R., Warne, J.P., Salas, E., Xu, A.W., and Debnath, J. (2015). Loss of Atg12, but not Atg5, in pro-opiomelanocortin neurons exacerbates diet-induced obesity. Autophagy *11*, 145–154. https://doi.org/10.1080/15548627.2014.998917.
16. Sobreira, N., Schiettecatte, F., Valle, D., and Hamosh, A. (2015). GeneMatcher: A Matching Tool for Connecting Investigators with an Interest in the Same Gene. Hum. Mutat. *36*, 928–930. https://doi.org/10.1002/humu.22844.
17. Li, H., and Durbin, R. (2009). Fast and accurate short read alignment with Burrows–Wheeler transform. Bioinformatics *25*, 1754–1760. https://doi.org/10.1093/bioinformatics/btp324.
18. McLaren, W., Gil, L., Hunt, S.E., Riat, H.S., Ritchie, G.R.S., Thormann, A., Flicek, P., and Cunningham, F. (2016). The Ensembl Variant Effect Predictor. Genome Biol. *17*, 122. https://doi.org/10.1186/s13059-016-0974-4.

19. McKenna, A., Hanna, M., Banks, E., Sivachenko, A., Cibulskis, K., Kernytsky, A., Garimella, K., Altshuler, D., Gabriel, S., Daly, M., and DePristo, M.A. (2010). The Genome Analysis Toolkit: A MapReduce framework for analyzing next-generation DNA sequencing data. Genome Res. *20*, 1297–1303. https://doi.org/10.1101/gr.107524.110.
20. DePristo, M.A., Banks, E., Poplin, R., Garimella, K.V., Maguire, J.R., Hartl, C., Philippakis, A.A., del Angel, G., Rivas, M.A., Hanna, M., et al. (2011). A framework for variation discovery and genotyping using next-generation DNA sequencing data. Nat. Genet. *43*, 491–498. https://doi.org/10.1038/ng.806.
21. Vigeland, M.D., Gjøtterud, K.S., and Selmer, K.K. (2016). FILTUS: a desktop GUI for fast and efficient detection of disease-causing variants, including a novel autozygosity detector. Bioinformatics *32*, 1592–1594. https://doi.org/10.1093/bioinformatics/btw046.
22. Kircher, M., Witten, D.M., Jain, P., O'Roak, B.J., Cooper, G.M., and Shendure, J. (2014). A general framework for estimating the relative pathogenicity of human genetic variants. Nat. Genet. *46*, 310–315. https://doi.org/10.1038/ng.2892.
23. Karczewski, K.J., Francioli, L.C., Tiao, G., Cummings, B.B., Alföldi, J., Wang, Q., Collins, R.L., Laricchia, K.M., Ganna, A., Birnbaum, D.P., et al. (2020). The mutational constraint spectrum quantified from variation in 141,456 humans. Nature *581*, 434–443. https://doi.org/10.1038/s41586-020-2308-7.
24. Richards, S., Aziz, N., Bale, S., Bick, D., Das, S., Gastier-Foster, J., Grody, W.W., Hegde, M., Lyon, E., Spector, E., et al. (2015). Standards and guidelines for the interpretation of sequence variants: a joint consensus recommendation of the American College of Medical Genetics and Genomics and the Association for Molecular Pathology. Genet. Med. *17*, 405–424. https://doi.org/10.1038/gim.2015.30.
25. Tavtigian, S.V., Harrison, S.M., Boucher, K.M., and Biesecker, L.G. (2020). Fitting a naturally scaled point system to the ACMG/AMP variant classification guidelines. Hum. Mutat. *41*, 1734–1737. https://doi.org/10.1002/humu.24088.
26. Metlagel, Z., Otomo, C., Takaesu, G., and Otomo, T. (2013). Structural basis of ATG3 recognition by the autophagic ubiquitin-like protein ATG12. Proc. Natl. Acad. Sci. USA *110*, 18844–18849. https://doi.org/10.1073/pnas.1314755110.
27. Bateman, A., Martin, M.-J., Orchard, S., Magrane, M., Agivetova, R., Ahmad, S., Alpi, E., Bowler-Barnett, E.H., Britto, R., Bursteinas, B., et al. (2021). UniProt: the universal protein knowledgebase in 2021. Nucleic Acids Res. *49*, D480–D489. https://doi.org/10.1093/nar/gkaa1100.
28. Evans, R., O'Neill, M., Pritzel, A., Antropova, N., Senior, A., Green, T., Žídek, A., Bates, R., Blackwell, S., Yim, J., et al. (2022). Protein complex prediction with AlphaFold-Multimer. Preprint at bioRxiv. https://doi.org/10.1101/2021.10.04.463034.
29. Luhr, M., Szalai, P., and Engedal, N. (2018). The lactate dehydrogenase sequestration assay — A simple and reliable method to determine bulk autophagic sequestration activity in mammalian cells. J. Vis. Exp. *2018*, e57971. https://doi.org/10.3791/57971.
30. Engedal, N., Luhr, M., Szalai, P., and Seglen, P.O. (2019). Measurement of bulk autophagy by a cargo sequestration assay. In Methods in Molecular Biology (Humana Press Inc.), pp. 307–313. https://doi.org/10.1007/978-1-4939-8873-0_20.
31. Ran, F.A., Hsu, P.D., Wright, J., Agarwala, V., Scott, D.A., and Zhang, F. (2013). Genome engineering using the CRISPR-Cas9 system. Nat. Protoc. *8*, 2281–2308. https://doi.org/10.1038/nprot.2013.143.
32. Yim, W.W.-Y., Yamamoto, H., and Mizushima, N. (2022). A pulse-chasable reporter processing assay for mammalian autophagic flux with HaloTag. eLife *11*, e78923. https://doi.org/10.7554/eLife.78923.
33. Cheong, H., and Klionsky, D.J. (2008). Biochemical Methods to Monitor Autophagy-Related Processes in Yeast. In Methods in Enzymology, pp. 1–26. https://doi.org/10.1016/S0076-6879(08)03201-1.
34. Noda, T., and Klionsky, D.J. (2008). The Quantitative Pho8Δ60 Assay of Nonspecific Autophagy. Methods Enzymol. *451*, 33–42. https://doi.org/10.1016/S0076-6879(08)03203-5.
35. Xie, Z., Nair, U., and Klionsky, D.J. (2008). Atg8 controls phagophore expansion during autophagosome formation. Mol. Biol. Cell *19*, 3290–3298. https://doi.org/10.1091/mbc.e07-12-1292.
36. Huang, W.-P., Scott, S.V., Kim, J., and Klionsky, D.J. (2000). The Itinerary of a Vesicle Component, Aut7p/Cvt5p, Terminates in the Yeast Vacuole via the Autophagy/Cvt Pathways. J. Biol. Chem. *275*, 5845–5851. https://doi.org/10.1074/jbc.275.8.5845.
37. Varshney, G.K., Pei, W., LaFave, M.C., Idol, J., Xu, L., Gallardo, V., Carrington, B., Bishop, K., Jones, M., Li, M., et al. (2015). High-throughput gene targeting and phenotyping in zebrafish using CRISPR/Cas9. Genome Res. *25*, 1030–1042. https://doi.org/10.1101/gr.186379.114.
38. Varshney, G.K., Carrington, B., Pei, W., Bishop, K., Chen, Z., Fan, C., Xu, L., Jones, M., LaFave, M.C., Ledin, J., et al. (2016). A high-throughput functional genomics workflow based on CRISPR/Cas9-mediated targeted mutagenesis in zebrafish. Nat. Protoc. *11*, 2357–2375. https://doi.org/10.1038/nprot.2016.141.
39. Lin, S.-J., Huang, K., Petree, C., Qin, W., Varshney, P., and Varshney, G.K. (2025). Optimizing gRNA selection for high-penetrance F0 CRISPR screening for interrogating disease gene function. Nucleic Acids Res. *53*, gkaf180. https://doi.org/10.1093/nar/gkaf180.
40. Kaiyrzhanov, R., Rad, A., Lin, S.-J., Bertoli-Avella, A., Kallemeijn, W.W., Godwin, A., Zaki, M.S., Huang, K., Lau, T., Petree, C., et al. (2024). Bi-allelic ACBD6 variants lead to a neurodevelopmental syndrome with progressive and complex movement disorders. Brain *147*, 1436–1456. https://doi.org/10.1093/brain/awad380.
41. Efthymiou, S., Leo, C.P., Deng, C., Lin, S.-J., Maroofian, R., Lin, R., Karagoz, I., Zhang, K., Kaiyrzhanov, R., Scardamaglia, A., et al. (2025). Bi-allelic pathogenic variants in TRMT1 disrupt tRNA modification and induce a neurodevelopmental disorder. Am. J. Hum. Genet. *112*, 1117–1138. https://doi.org/10.1016/j.ajhg.2025.03.015.
42. Makrythanasis, P., Maroofian, R., Stray-Pedersen, A., Musaev, D., Zaki, M.S., Mahmoud, I.G., Selim, L., Elbadawy, A., Jhangiani, S.N., Coban Akdemir, Z.H., et al. (2018). Biallelic variants in KIF14 cause intellectual disability with microcephaly. Eur. J. Hum. Genet. *26*, 330–339. https://doi.org/10.1038/s41431-017-0088-9.
43. Graham, C., Stansfield, P., and Chris, R. (2023). Conservation-Colab: Conservation to 3D structure Colab v1.0.2 (Zenodo). https://doi.org/10.5281/zenodo.10062701.
44. Otomo, C., Metlagel, Z., Takaesu, G., and Otomo, T. (2013). Structure of the human ATG12~ATG5 conjugate required for LC3 lipidation in autophagy. Nat. Struct. Mol. Biol. *20*, 59–66. https://doi.org/10.1038/nsmb.2431.

45. Mauvezin, C., and Neufeld, T.P. (2015). Bafilomycin A1 disrupts autophagic flux by inhibiting both V-ATPase-dependent acidification and Ca-P60A/SERCA-dependent autophagosome-lysosome fusion. Autophagy *11*, 1437–1438. https://doi.org/10.1080/15548627.2015.1066957.
46. Liebl, M.P., Meister, S.C., Frey, L., Hendrich, K., Klemmer, A., Wohlfart, B., Untucht, C., Nuber, J., Pohl, C., and Lakics, V. (2022). Robust LC3B lipidation analysis by precisely adjusting autophagic flux. Sci. Rep. *12*, 79. https://doi.org/10.1038/s41598-021-03875-8.
47. Mizushima, N. (2025). Autophagic flux measurement: Cargo degradation versus generation of degradation products. Curr. Opin. Cell Biol. *93*, 102463. https://doi.org/10.1016/j.ceb.2025.102463.
48. Katsuragi, Y., Ichimura, Y., and Komatsu, M. (2015). p62/SQSTM1 functions as a signaling hub and an autophagy adaptor. FEBS J. *282*, 4672–4678. https://doi.org/10.1111/febs.13540.
49. Sou, Y.s., Waguri, S., Iwata, J.i., Ueno, T., Fujimura, T., Hara, T., Sawada, N., Yamada, A., Mizushima, N., Uchiyama, Y., et al. (2008). The Atg8 conjugation system is indispensable for proper development of autophagic isolation membranes in mice. Mol. Biol. Cell *19*, 4762–4775. https://doi.org/10.1091/mbc.e08-03-0309.
50. Tsukamoto, S., Kuma, A., Murakami, M., Kishi, C., Yamamoto, A., and Mizushima, N. (2008). Autophagy Is Essential for Preimplantation Development of Mouse Embryos. Science *321*, 117–120. https://doi.org/10.1126/science.1154822.
51. Millen, K.J., and Gleeson, J.G. (2008). Cerebellar Development and Disease. Curr. Opin. Neurobiol. *18*, 12–19. https://doi.org/10.1016/J.CONB.2008.05.010.
52. McWilliams, T.G., Prescott, A.R., Allen, G.F.G., Tamjar, J., Munson, M.J., Thomson, C., Muqit, M.M.K., and Ganley, I.G. (2016). mito -QC illuminates mitophagy and mitochondrial architecture in vivo. J. Cell Biol. *214*, 333–345. https://doi.org/10.1083/jcb.201603039.
53. Rappe, A., Vihinen, H.A., Suomi, F., Hassinen, A.J., Ehsan, H., Jokitalo, E.S., and McWilliams, T.G. (2024). Longitudinal autophagy profiling of the mammalian brain reveals sustained mitophagy throughout healthy aging. EMBO J. *43*, 6199–6231. https://doi.org/10.1038/s44318-024-00241-y.
54. Hosokawa, N., Hara, Y., and Mizushima, N. (2006). Generation of cell lines with tetracycline-regulated autophagy and a role for autophagy in controlling cell size. FEBS Lett. *580*, 2623–2629. https://doi.org/10.1016/j.febslet.2006.04.008.
55. Haack, T.B., Hogarth, P., Gregory, A., Prokisch, H., and Hayflick, S.J. (2013). BPAN: the only X-linked dominant NBIA disorder. Int. Rev. Neurobiol. *110*, 85–90. https://doi.org/10.1016/B978-0-12-410502-7.00005-3.
56. Shimizu, T., Tamura, N., Nishimura, T., Saito, C., Yamamoto, H., and Mizushima, N. (2023). Comprehensive analysis of autophagic functions of WIPI family proteins and their implications for the pathogenesis of β-propeller associated neurodegeneration. Hum. Mol. Genet. *32*, 2623–2637. https://doi.org/10.1093/hmg/ddad096.
57. Collier, J.J., Suomi, F., Oláhová, M., McWilliams, T.G., and Taylor, R.W. (2021). Emerging roles of ATG7 in human health and disease. EMBO Mol. Med. *13*, EMMM202114824. https://doi.org/10.15252/emmm.202114824.

The American Journal of Human Genetics, Volume *113*

Supplemental information

Bi-allelic *ATG12* variants impair autophagy and cause a neurodevelopmental disorder

James Lambton, Shotaro Asano, Yuxiang Huang, Fumi Suomi, Tomoya Eguchi, Cassidy Petree, Kevin Huang, Magali Prigent, Aliza Imam, Thomas J. McCorvie, Daniel Warren, Emma Hobson, Helen McCullagh, Doriana Misceo, Anna Bjerre, Marie F. Smeland, Claus Klingenberg, Eirik Frengen, Swati Naik, Gavin Ryan, Annapurna Sudarsanam, Katherine Foster, Pradeep Vasudevan, Rajib Samanta, Fatima Rahman, Shazia Maqbool, Vrajesh Udani, Stephanie Efthymiou, Henry Houlden, Robert McFarland, Jack J. Collier, Reza Maroofian, Wyatt W. Yue, Gaurav K. Varshney, Daniel J. Klionsky, Renaud Legouis, Thomas G. McWilliams, Noboru Mizushima, Monika Oláhová, Charlotte L. Alston, and Robert W. Taylor

Supplemental materials

Clinical Descriptions

Family 1

S1 (Family 1, **Figure 1A**), a female infant, was the first child born to non-consanguineous white European parents following *in vitro* fertilization (IVF) for unexplained infertility. Born at term, by normal vaginal delivery following an uneventful pregnancy, she did not require resuscitation and weighed 3.225 kg (25th-50th centile). Newborn examination was normal with the exception of overlapping 4th and 5th toes bilaterally. On day 3 of life, feeding difficulties, excessive weight loss and jaundice were noted. Examination revealed hypotonia and apnoea when feeding. Supplemental oxygen and nasogastric tube feeding were commenced, and a subsequent swallowing assessment confirmed a poorly coordinated swallow.

Axial hypotonia with excessive head lag remained a prominent feature on neurological review at 6 weeks of age when myopathic facies and a head circumference of 36 cm (9-25th centile) were also recorded. By 2 months of age, limb movements were markedly decreased, and she was unable to fix and follow. Social smile was lost at 7 months. In addition, a clinical diagnosis of infantile spasms was supported by a hypsarrhythmic electroencephalogram (EEG) and anti-epileptic medications were commenced. Cranial magnetic resonance imaging (MRI) performed at 4 weeks and 7 months revealed diffuse parenchymal volume loss involving both grey and white matter (**Figure 1B**). The corpus callosum was hypoplastic with marked volume loss of cerebellar hemispheres and vermis at 7 months compared to 4 weeks. The mamillary bodies were absent and the hippocampi simplified with delayed myelination. S1 died aged 12 months due to her rapidly progressive neurodegenerative condition with refractory epilepsy.

Further clinical review by paediatric neurology led to a range of neurological, metabolic, genetic and radiological investigations that were diagnostically uninformative. Blood and fibroblasts were taken for cytogenetic (array CGH) and molecular genetic analysis.

S2, a male infant and younger sibling of S1, was born at 38+5 weeks gestation by normal vaginal delivery and weighed 3.615 kg (50th–75th centile) with a head circumference of 35.4 cm (25th-50th centile). No resuscitation was required at birth, but hypotonia and paucity of movement was noted. He had bilateral positional talipes with overlapping 4th and 5th toes on the right foot. Tachypnoea with low oxygen saturation prompted admission to the neonatal

intensive care unit where he was initially treated for transient tachypnoea of the newborn. A persistent oxygen requirement and feeding difficulties prompted further medical investigations.

At 2 weeks of age, recurrent seizures were treated with levetiracetam. Neurological examination revealed nystagmus, reduced anti-gravity movements and hypotonia. A cranial MRI at 11 days of age demonstrated a structural malformation with deep sulcation of the right occipital lobe and generalised (particularly vermian) cerebellar and corpus callosal hypoplasia. A comparison of S1 and S2 scans suggested a number of common features, most notably the vermian volume loss and callosal hypoplasia.

Ongoing feeding difficulties prompted insertion of a gastrostomy at 4 months of age. Around this time increased seizure activity was noted and while initial EEGs had been unremarkable, by 4 months the EEG was consistent with epileptic encephalopathy. Blood and fibroblasts were taken for cytogenetic (array CGH) and molecular genetic analysis. S2 died at 6 months of age following a presumed viral infection and subsequent increase in apnoeic episodes. The cause of death was recorded as a neurodegenerative condition with refractory epilepsy as in the case of S1, the older sibling.

Family 2

S3, a male, is the fourth child of healthy consanguineous, first cousin parents originating from the Middle East (Family 2, **Figure 1A**). His two oldest siblings are healthy. One older sister had difficulty with walking and speaking, impaired vision with nystagmus and died at the age of 7 years from end-stage kidney disease (ESKD). Many of these features were similar to S3, suggesting she may have had the same disease as S3; however no genetic analyses were performed. His mother also reported three previous pregnancy losses.

Pregnancy, term delivery and the neonatal period of S3 were normal. Limited detail is available on his early life, but psychomotor delay became evident during the first year and by 3 years he had experienced a single seizure though did not go on to develop epilepsy.

At 4 years of age, he was diagnosed with kidney disease, anaemia and thrombocytopenia. A clearly defined renal diagnosis has never been applied, but he subsequently developed ESKD. He received peritoneal dialysis from 8 until the age of 13 years when a renal transplant was performed. Renal ultrasound and renal MRI before the transplant showed bilateral atrophic kidneys with small cysts.

At 9 years of age, he was diagnosed with pancreatitis and cholecystitis, subsequently having a cholecystectomy. At 12 years of age, he also had surgical repair for large bilateral inguinal hernias. He was severely growth retarded with Z-score for length at -6 SD before the renal transplant. Growth remained impaired and did not improve substantially after transplantation. He has been diagnosed with hypothyroidism and receives thyroxine replacement therapy.

He has limited verbal language skills, moderate intellectual disability and wears hearing aids to assist with bilateral sensorineural hearing impairment. He walks only with the aid of a walker due to ataxia. He has had dry and scaly skin since early childhood. Brain MRI obtained at 12 years of age showed atrophy of both cerebellar hemispheres and the vermis and widespread bilateral polymicrogyria (**Figure 1B)**. An ophthalmological examination revealed bilateral optic atrophy and typical Bull's eye maculopathy.

His clinical appearance in adolescence is one of short stature, with a slender build. He has microcephaly, down slanting almond-shaped eyes and blepharophimosis. He has a broad nasal bridge, anteverted nostrils, long philtrum and cupid's bow upper lip. His ears are low set with an irregular helix, and he has sparse scalp hair (**Figure 1C**). He also has bilateral fifth finger clinodactyly of both hands.

Family 3

S4 is the first child of consanguineous parents of Indian heritage from the Daman region, India (Family 3, **Figure 1A-C**). He was born by lower segment cesarean section (birth weight of 2.88kg and HC of 34 cm) following a pregnancy complicated by gestational diabetes mellitus. An antenatal diagnosis of dilated urinary bladder was not confirmed on postnatal abdominal ultrasound scan, which was reported normal. Parents believed that he was well until concerns were raised at a six-week check by the health visitor who felt that he was not fixing and following. At about 3 months old he experienced his first seizure, which lasted for more than 45 minutes. Currently, he is on levetiracetam, vigabatrin and gabapentin along with the ketogenic diet. S4 has a younger brother who is now 3.5 months old and is well.

Family 4

S5 is a male child of healthy non-consanguineous parents (Family 4, **Figure 1A, C**). The proband's 6-year-old brother died of atypical brainstem glioma. The proband has one sister and she is well.

Pregnancy with S5 followed a natural conception and proceeded without incident until a fetal ultrasound scan at approximately 20 weeks gestation revealed talipes and dilated ventricles. The pregnancy was then monitored and S5 was born at 39 weeks gestation with a normal birth weight (3095g). His birth length was 47 cm and birth head circumference was 33cm. He was noted to have neonatal jaundice, poor feeding and tongue tie. He received phototherapy and was discharged home after 3 days. He was readmitted soon afterwards with abnormal movements. Although EEG was reported normal, he went on to have clinical seizures and brain MRI scan revealed dysgenesis of the corpus callosum, prominent cavum septum pellucidum, abnormal cerebellar vermis, small cerebellar hemispheres and a small posterior fossa (**Figure 1B**). In view of the above features, microarray analysis was requested which did not find any copy number variation. Rapid WES was then requested under R14 category as per national genomic test directory and the result was normal. Metabolic investigations including blood lactate, organic acids, amino acids, carnitine, very long chain fatty acids were normal. The child subsequently exhibited central hypotonia, microcephaly, significant global developmental delay, epileptic encephalopathy, cerebral irritability and movement disorder with dystonia and dyskinesia. Whole genome sequencing (WGS) was requested as a trio analysis with R27, R59, R57, R98, R69 panels as per national genomic test directory and the result was also normal.

Clinical review at 4 years 6 months showed significant hypotonia, profound developmental delay with a non-verbal and non-mobile child who has a gastrostomy *in situ*, intractable epilepsy and a movement disorder. Palliative care has been involved.

Family 5

S6 is male and the third child of consanguineous Pakistani parents. He presented at 7 months of age with concerns regarding developmental delay, failure to thrive and seizures from 1 month of age. Seizures were controlled by one year of age with levetiracetam 40mg/kg/day. Antenatal and post-natal history were not significant. He was born at term via spontaneous vaginal delivery. He was partially vaccinated and had multiple hospital admissions with uncontrolled seizures. An older brother had impaired vision with borderline intellectual disability, while an older sister had a history of urethral meatal stenosis but no developmental delay. A younger sister had multiple café au lait macules, global developmental delay, behavioural issues (aggression, hyperactivity) and recurrent chest and gastrointestinal infections.

On examination at age 7 months, occipitofrontal circumference (OFC) was 37.5cm, weight 4.5kg with clinical signs of malnutrition but no obvious dysmorphic features. He was floppy with poor truncal control, head lag and hyporeflexia. He appeared unable to grasp objects placed in his palms. He could fix on familiar faces but was unable to follow. Eye examination revealed vernal keratoconjunctivitis with bilateral pseudogerontoxon. Right eye had giant and limbal papillae while upper tarsal micropapillae were observed in left eye. Over time, he developed spasticity with failure to gain weight. Speech did not progress beyond simple vocalisations. EEG at 7 months of life was normal but no neuroimaging was obtained. At 2.5 years he was emaciated, weighing only 2kg (<0.4th centile), and passed away following a period of vomiting leading to severe dehydration.

Supplemental Figures

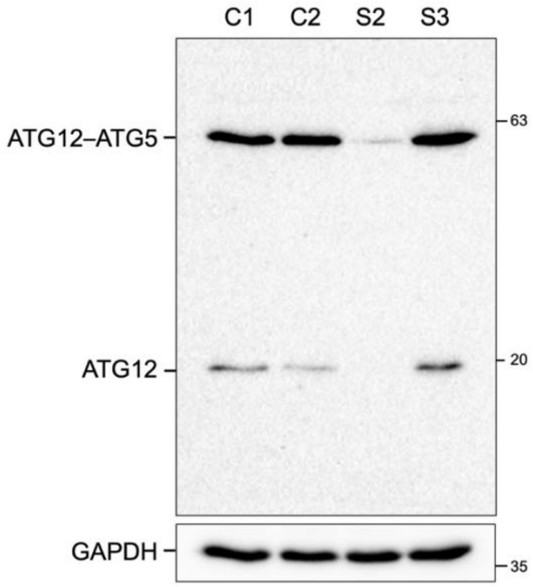

Figure S1. Full length western blot analysis of ATG12 species. Western blot analysis of patient derived fibroblasts produces two distinct bands corresponding to the ATG12-ATG5 heterodimer, and unconjugated ATG12. The c.363+3A>T *ATG12* variant observed in S2 does not generate any detectable additional ATG12 species.

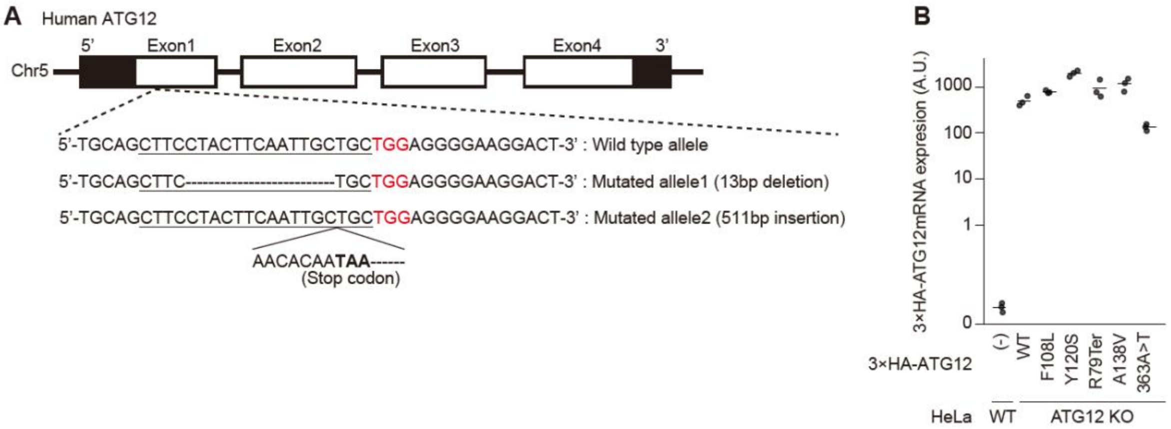

Figure S2. Generation of ATG12 knockout cells and *ATG12* mRNA expression of ATG12 variants. (**A**) Generation of ATG12 knockout (KO) cells. Schematic representation of the Cas9-gRNA-target site (underlined) in the *ATG12* genomic locus. The protospacer-adjacent motif sequence is shown in red. A deletion of 13 bp and an insertion of 511 bp in the knockout allele are indicated. **(B)** Expression levels of *ATG12* variants. The mRNA expression levels of each *ATG12* variant in control (HeLa WT) cells and ATG12-KO HeLa cells expressing the indicated constructs were quantified by qPCR. Transcripts derived from the 3×HA-tagged ATG12 constructs were detected using primers specific for the 3×HA tag (forward, TTCCTGACTATGCGGGCTAT; reverse, CAGCGTAATCTGGAACGTCATA). Relative expression levels were calculated using the ΔΔCt method and normalized to ActB, detected with the following primers (forward, CCTTCTTGGGTATGGAATCCTGT; reverse, CACTGTGTTGGCATAGAGGTCTTTAC).

Table S1. Primers used for genomic DNA confirmation of *ATG12* variants

Target	Primer sequence
ATG12_ex3F	GAAAGTCATCTTTTCTACCCCAGA
ATG12_ex3R	TTGTGGGTAAGTGGGGACAT
ATG12_Family2 F	ACAAGTAGGAGCAATGGGTGT
ATG12_Family 2 R	AGCTCATTCATGCCTAAGATGCT

Table S2. Primers used for cDNA investigations into *ATG12* splice variant.

Target	Primer sequence
ATG12_cDNA_F	GGATGTCTCCCCCAGAAACAA
ATG12_cDNA_R	AACAAGTAGGAGCAATGGGTGT

Table S3. Genotype and source of *S. cerevisiae* used in this study.

Strain	Genotype	Source	Purpose
JH914	SEY6210+*atg12Δ::KANMX6 PGK1-GFP::natMX6*	This study	Pgk1-GFP assay
JH947	WLY176 *pRS405-ATG8p-GFP-ATG8::LEU2 atg12Δ::HIS3MX6 pRS406-ATG12p-3xHA-ATG12 F154L::URA3*	This study	Atg8 lipidation, GFP-Atg8, and Pho8Δ60 assays
JH948	WLY176 *pRS405-ATG8p-GFP-ATG8::LEU2 atg12Δ::HIS3MX6 pRS406-ATG12p-3xHA-ATG12 W166S::URA3*	This study	Atg8 lipidation, GFP-Atg8, and Pho8Δ60 assays
JH949	SEY6210+*atg12Δ::KANMX6 PGK1-GFP::natMX6 pRS406::URA3*	This study	Pgk1-GFP assay
JH950	SEY6210+*atg12Δ::KANMX6 PGK1-GFP::natMX6 pRS406-ATG12p-3xHA-ATG12::URA3*	This study	Pgk1-GFP assay
JH952	SEY6210+*atg12Δ::KANMX6 PGK1-GFP::natMX6 pRS406-ATG12p-3xHA-ATG12 F154L::URA3*	This study	Pgk1-GFP assay
JH953	SEY6210+*atg12Δ::KANMX6 PGK1-GFP::natMX6 pRS406-ATG12p-3xHA-ATG12 W166S::URA3*	This study	Pgk1-GFP assay
JH954	WLY176 *pRS405-ATG8p-GFP-ATG8::LEU2 atg12Δ::HIS3MX6 pRS406-ATG12p-3xHA-ATG12 A184V::URA3*	This study	Atg8 lipidation, GFP-Atg8, and Pho8Δ60 assays
JH955	SEY6210+*atg12Δ::KANMX6 PGK1-GFP::natMX6 pRS406-ATG12p-3xHA-ATG12 A184V::URA3*	This study	Pgk1-GFP assay
OC751	BY4742, *atg12::KanMX4, ura3Δ0, his3Δ1, leu2Δ0, lys2Δ0*	YKO collection	GFP-Atg8 *in-vivo* microscopy
OC588	BY4742, *atg8::GFP-ATG8-URA3, ura3Δ0, his3Δ1, leu2Δ0, lys2Δ0*	This study	GFP-Atg8 *in-vivo* microscopy
OC744	BY4742, *atg8::GFP-ATG8-URA3, atg12::KanMX4, ura3Δ0, his3Δ1, leu2Δ0, lys2Δ0*	This study	GFP-Atg8 *in-vivo* microscopy
YLY290	WLY176 *pRS405-ATG8p-GFP-ATG8::LEU2 atg12Δ::HIS3MX6 pRS406::URA3*	This study	Atg8 lipidation, GFP-Atg8, and Pho8Δ60 assays
YLY291	WLY176 *pRS405-ATG8p-GFP-ATG8::LEU2 atg12Δ::HIS3MX6 pRS406-ATG12p-3xHA-ATG12::URA3*	This study	Atg8 lipidation, GFP-Atg8, and Pho8Δ60 assays

Table S4. Oligonucleotides used to generate plasmid constructs for yeast complementation studies.

Target	Primer sequence
ATG12 Cloning	5' AGTCTAGAACCTAGTCTTCCATTTACATCG 3'
	5' AACTGCAGGGGTCTTTGGTGAAAAAGGG 3'
ATG12 W166S mutagenesis	5' TTGGTGAACTTTCGATGCAATTCAAGAC 3'
	5' TATTTTGCTGCGGACTTGGC 3'

ATG12 A184V	5’ TTGTGCATCCGTAGTGTTTGGTTAAATAC 3’
	5’ TAACTTACAATAAGCTCATCATTAGTC 3’

Table S5. Overview of phenotypes associated with described congenital disorders in *ATG* genes.

**Gene**	***ATG12***						***ATG7***	***ATG5***
**No. of individuals**	***n* = 6**						***n* = 12**	***n* = 2**
**Reference**	**Lambton *et al.* present study**						**Collier *et al* 2021[8]**	**Yapici *et al* 2005[10] Kim *et al* 2016[9]**
Subject	S1	S2	S3	S4	S5	S6	S1-12	Subject 1 and 2[a]
Age at last examination/ death	Died 12 m	4 m/died 6 m	17 y	6 y	4 y 6 m / died 5 y	7 m / died 2 y 6 m	(1-71 y)	(13 y and 15 y)
Ataxia	NA	NA	+ walks with support	Nonambulant	NA	Did not achieve walking	9/11	2/2
DD	+	+	+	+	+	+	12/12	2/2
Intellectual disability	+ EE	+ EE	+	+	+ EE	+	12/12	2/2
Seizures	+ IS	+	+	+ IS	+	+	2/12	0/2
CC hypoplasia	+	+	-	+	+(Body of cc thin and absent posteriorly)	N/A	7/9	0/2
Cerebellar (vermian) hypoplasia	+	+	+	+	+	N/A	7/11	2/2
Other brain anomaly	Reduced white and grey matter	Deep sulcation occipital lobe	Polymicrogyria (widespread)	Microcephaly with brachycephaly & cerebellocerebral atrophy, thinning of pons, brainstem. hypomyelination	Prominent cavum septum pellucidum, small cerebellar hemispheres, small posterior fossa	N/A	Brain volume loss 3/9	-

Sensorineural deafness	NA	NA	+	NA	NA	N/A	2/12	NA
Optic atrophy	NA	-	+	+	NA	No but vernal keratoconjunctivitis with bilateral pseudogerontoxon	7/9	NA
Kidney disease	NA	NA	ESKD	NA	-	-	NA	NA
Dysmorphology	NA	NA	+	+	+	-	4/12	NA
Growth retardation	NA	NA	+	+	+	+	3/8	NA
Other			Dry scaly skin, sparse hair, pancreatitis, cholecystitis, inguinal hernia, hypothyreosis		Movement disorder with dystonia and dyskinesia, cerebral irritability, PEG fed	Repeated chest and GI infections, failure to thrive, microcephaly		Club feet 1/2

+ = present; - = absent; CC = corpus callosum; CI = cognitive impairment; DD= developmental delay; EE = epileptic encephalopathy; ESKD = end-stage kidney disease; IS = infantile spasms; m = months, NA = not assessed; S = subject; y = years; PEG = percutaneous endoscopic gastrostomy. [a] Subject 1 and Subject 2 from family 1 in Yapici *et al* 2005; reassessed as subjects 601 and 602 in Kim *et al* 2016.

Further acknowledgments

We are grateful to the families for participating to the study. We thank Dr. Siren Berland and co-workers at Haukeland Universitetssjukehus, Bergen, Norway, for performing genetic testing in a clinical setting. We thank the staff of the HiLIFE electron microscopy unit (directed by Dr. Eija Jokitalo) for their excellent technical assistance, and Dr. Helena Vihinen for insightful advice on sample preparation. We are grateful to Professor Eeva-Liisa Eskilinen (University of Turku, FI) for insightful discussions and guidance on discriminating autophagic sub-compartments. We thank Yongheng Liang (Nanjing Agricultural University) for plasmid *pP1kGFP-ATG8(406)* and Florence Piron Prunier for technical advice. We also thank UNINETT Sigma2, the National Infrastructure for High Performance Computing and Data Storage in Norway, for support to store and analyse High-Throughput Sequencing data.
